# Synthesis and analytical characterization of new thiazol-2-(*3H*)-ones as human neutrophil elastase (HNE) inhibitors

**DOI:** 10.1186/s13065-017-0358-1

**Published:** 2017-12-06

**Authors:** Letizia Crocetti, Gianluca Bartolucci, Agostino Cilibrizzi, Maria Paola Giovannoni, Gabriella Guerrini, Antonella Iacovone, Marta Menicatti, Igor A. Schepetkin, Andrei I. Khlebnikov, Mark T. Quinn, Claudia Vergelli

**Affiliations:** 1 Sezione di Farmaceutica e Nutraceutica, NEUROFARBA, Università degli Studi di Firenze, Via Ugo Schiff 6, 50019 Sesto Fiorentino, Firenze, Italy; 2 Institute of Pharmaceutical Science, King’s College London, 150 Stamford Street, London, SE1 9NH UK; 3 Department of Microbiology and Immunology, Montana State University, Bozeman, MT 59717 USA; 4 Department of Biotechnology and Organic Chemistry, Tomsk Polytechnic University, Tomsk, 634050 Russia; 5 Scientific Research Institute of Biological Medicine, Altai State University, Barnaul, 656049 Russia

**Keywords:** Thiazol-2-(*3H*)-one, Synthesis, LC–MS/MS, ERMS, Human neutrophil elastase

## Abstract

Human neutrophil elastase (HNE) is a potent serine protease belonging to the chymotrypsin family and is involved in a variety of pathologies affecting the respiratory system. Thus, compounds able to inhibit HNE proteolytic activity could represent effective therapeutics. We present here the synthesis of new thiazol-2-(*3H*)-ones as an elaboration of potent HNE inhibitors with an isoxazol-5-(*2H*)-one scaffold that we recently identified. Two-dimensional NMR spectroscopic techniques and tandem mass spectrometry allowed us to correctly assign the structure of the final compounds arising from both tautomers of the thiazol-2-(*3H*)-one nucleus (N-3 of the thiazol-2-(*3H*)-one and 3-OH of the thiazole). All new compounds were tested as HNE inhibitors, and no activity was found at the highest concentration used (40 µM), demonstrating that the thiazol-2-(*3H*)-one is not a good scaffold for HNE inhibitors. Molecular modelling experiments indicate that the low-energy pose might limit the nucleophilic attack on the endocyclic carbonyl group of the thiazolone-based compounds by HNE catalytic Ser195, in contrast to isoxazol-5-(*2H*)-one analogues.

**Electronic supplementary material:**

The online version of this article (10.1186/s13065-017-0358-1) contains supplementary material, which is available to authorized users.

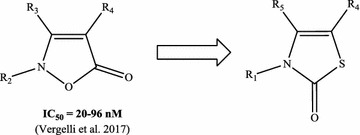

## Introduction

**Fig. 1:**
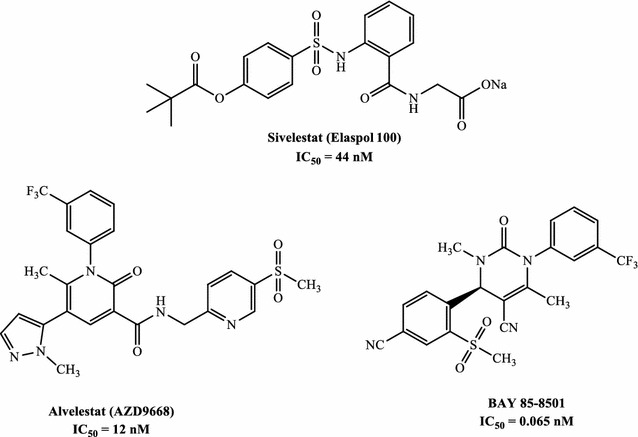
HNE inhibitors

Human neutrophil elastase (HNE) is a small, soluble glycoprotein of about 30 kDa belonging to the chymotrypsin family of serine proteases [1] and is expressed primarily in neutrophils, but also in monocytes and mast cells [2]. HNE plays an important role in the maintenance of tissue homeostasis and repair due to its proteolytic action on structural proteins [2, 3]. HNE performs its proteolytic action through a catalytic triad consisting of Ser195-Asp102-His57, where the powerful nucleophile oxygen of Ser195 attacks the carbonyl carbon involved in the peptide bond [4, 5]. HNE is involved in a variety of pathologies affecting the respiratory system, such as chronic obstructive pulmonary disease (COPD), acute respiratory distress syndrome (ARDS), acute lung injury (ALI) and cystic fibrosis (CF) [6–8]. Currently, Sivelestat, which is used for treatment of ALI and ARDS ([9], Fig. 1), and Prolastin, which is used in the therapy of α1-antitripsin deficiency (AATD) [10], are the only HNE inhibitors commercially available. Moreover, Alvelestat (AZD9668, Fig. 1) [11, 12] and Bay 85-8501 [13] are two promising compounds in phase II clinical trials for the treatment of COPD and CF (Fig. 1).

**Fig. 2:**
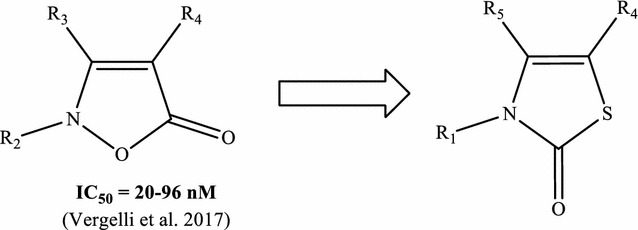
Reference isoxazolone compounds and new thiazolone derivatives

Our research group is involved in the design and synthesis of small molecules with HNE inhibitory activity, and we have identified a number of new classes of inhibitors based on different bicyclic scaffolds [14–17]. The most potent compounds are *N*-benzoylindazole derivatives, which have IC_50_ values in the low nanomolar range (IC_50_ = 7–80 nM) [14, 16]. Moreover, we recently reported new isoxazolone-based derivatives with HNE inhibitory activity in the low nanomolar range (IC_50_ = 20–96 nM) [18]. Molecular modeling studies on this class of compounds allowed to establish which carbonyl group was involved in the attack of Ser195 (i.e., HNE catalytic residue) and define the endocyclic carbonyl at position 5 of the isoxazolone *core* as a critical requirement for inhibitory activity [18]. Starting from these results, we report here the development and analytical characterization of a new series of heterocyclic compounds based on the thiazol-2-(3*H*)-one scaffold originally designed as possible HNE inhibitors (Fig. 2).

## Chemistry

**Scheme 1:**
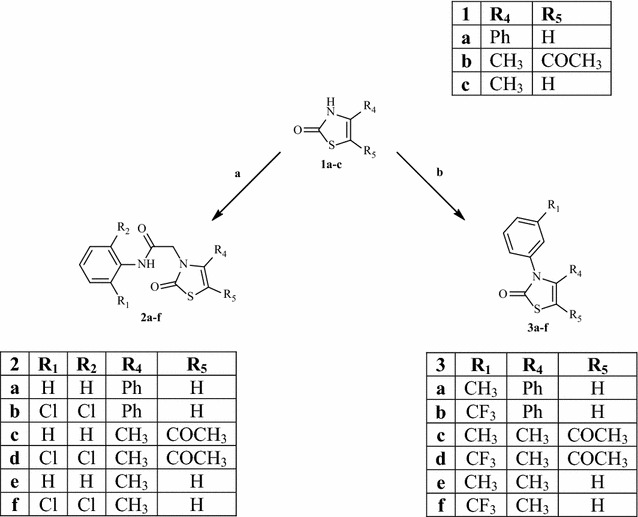
Reactions and conditions: **a** appropriate substituted 2-chloro-*N*-phenylacetamide, CH_3_CN anhydrous, K_2_CO_3_, 80 °C, 6–7 h; **b** appropriate (substituted)-phenylboronic acid, (CH_3_COO)_2_Cu, EtN_3_, CH_2_Cl_2_

**Scheme 2:**
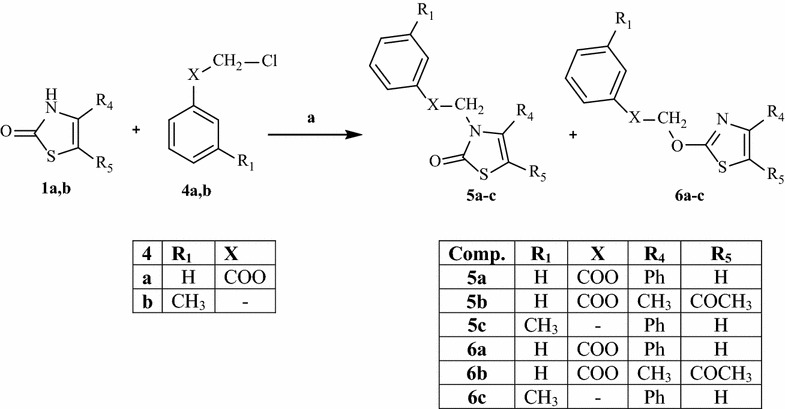
Reactions and conditions: **a** CH_3_CN anhydrous, K_2_CO_3_, 80 °C, 3–8 h

**Scheme 3:**
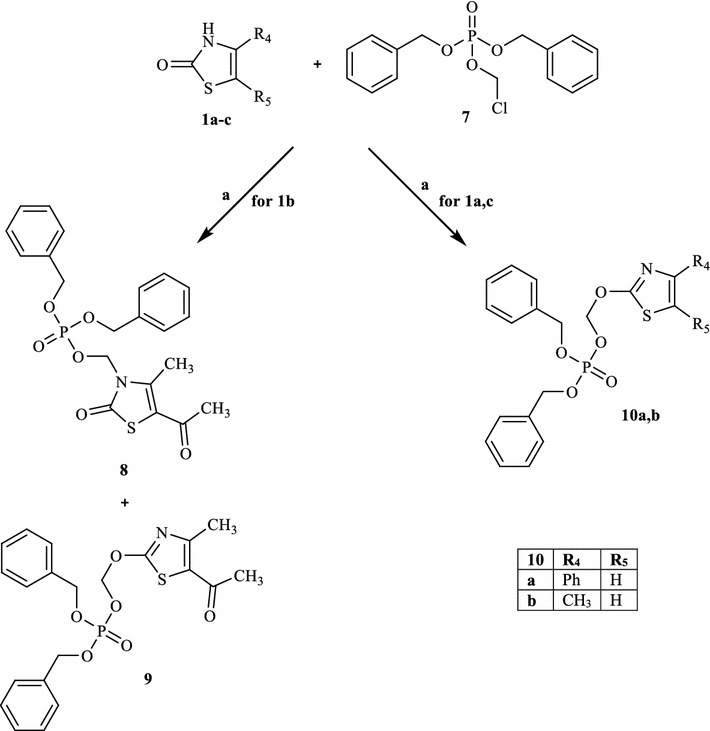
Reactions and conditions: **a** CH_3_CN anhydrous, K_2_CO_3_, 80 °C, 6 h

All compounds were synthesized as reported in Schemes 1, 2, 3, and the structures were univocally confirmed on the basis of analytical and spectral data, such as two-dimensional NMR spectroscopic techniques (i.e. HSQC and HMBC, see Additional file 1) and tandem mass spectrometry. According to literature on thiazol-2-(3*H*)-ones, the predominant isomer in solution seems to be the 2-oxo form [19], although the alkylation reactions can take place on both tautomers (i.e., N-3 of the thiazol-2-(3*H*)-one and 3-OH of the thiazole) [20].

Scheme 1 shows the synthetic pathways to obtain final compounds **2a**–**f**, and **3a**–**f**, which are all 3-N-derivatives. Products **2a**–**f** were obtained by standard alkylation on precursors **1a**–**c** [21–23], using appropriately substituted 2-chloro-*N*-phenylacetamides and K_2_CO_3_ in anhydrous acetonitrile at reflux. The N-aryl derivatives **3a**–**f** were obtained through a coupling reaction on **1a**–**c** with commercially available phenylboronic acids in the presence of EtN_3_ and (Ac)_2_Cu (Scheme 1). The reactions presented in Scheme 2 show the formation of both tautomers (**5a**–**c** and **6a**–**c**), although the N-alkylated derivatives are still the predominant compounds. Lastly, the introduction of a dibenzyl phosphate group on **1a**–**c** (Scheme 3) resulted in O-alkylated isomers (**10a**, **b**) when using compounds **1a** and **1c**, while the same reaction on **1b** led to both isomers **8** and **9** at a 2:1 ratio.

## Analytical characterization

**Fig. 3:**
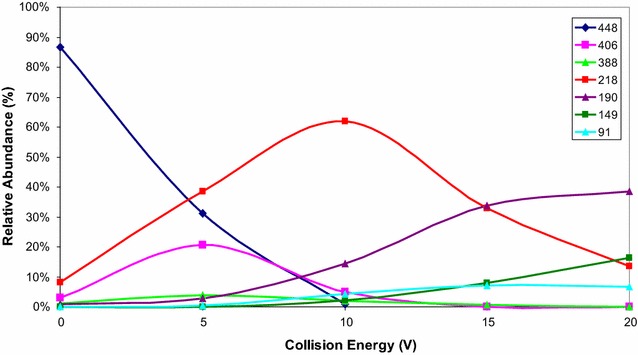
Breakdown curves obtained from ERMS experiment on the compound **8**

**Fig. 4:**
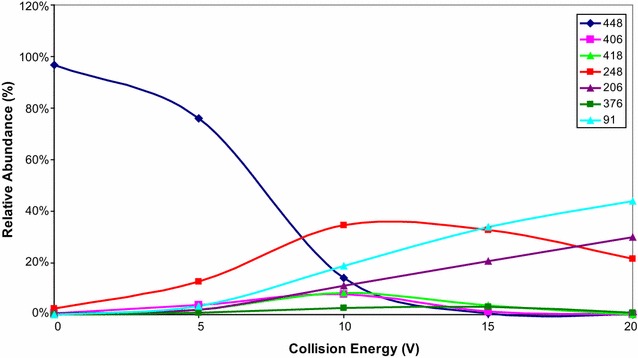
Breakdown curves obtained from ERMS experiment on the compound **9**

**Fig. 5:**
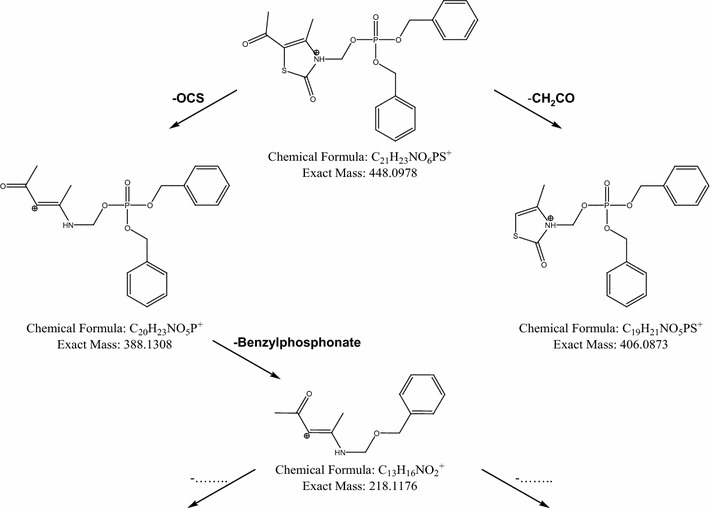
Proposed fragmentation pathway for compound **8**

**Fig. 6:**
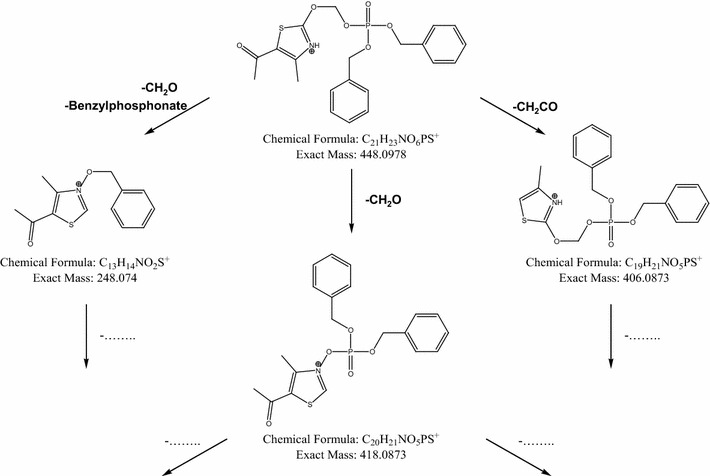
Proposed fragmentation pathway for compound **9**

The isomeric compounds **8** and **9** (Scheme 3) feature different positions for the dibenzyl phosphate moiety in their structure. Consequently, a series of MS/MS experiments applying different collision energies (ERMS) were used to characterize these compounds. The breakdown curves obtained from the [M+H]^+^ ion species of the two isomers are shown in Figs. 3 and 4. Comparison of these fragmentation patterns showed the formation of different product ions between the two isomers. Only the fragment at 406 m/z was common for both analytes, but its formation was reached at different collision energies (Figs. 3 and 4). On the basis of these results, the proposed fragmentation patterns of isomers **8** and **9** are presented in Figs. 5 and 6. As shown in Fig. 5, isomer **8** has two potential fragmentation pathways. The first pathway involves carbonyl sulfide loss, which is favored in heterocyclic moieties, followed by a rearrangement and loss of benzylphosphate (product ions 388 and 218 m/z, respectively). The second pathway involves loss of ethenone from the thiazolone ring (product ion 406 m/z).

As shown in Fig. 6, isomer **9** has at least three potential fragmentation ways. The first pathway involves loss of formaldehyde (product ion 418 m/z). The second pathway involves loss of formaldehyde, rearrangement, and loss of benzylphosphate (product ion 248 m/z). The final possible pathway involves loss of ethenone from the thiazolone ring (product ion 406 m/z). Therefore, it is possible that, under MS/MS conditions, isomer **8** represents the *N*-benzylphosphate derivative, leaving the possibility that the carbonyl sulfide could be lost from heterocyclic ring. In contrast, formation of isomer **9** suggests that formaldehyde loss is preferred, resulting in occupation of the heterocyclic ring oxygen (O-benzylphosphate isomer).

**Fig. 7:**
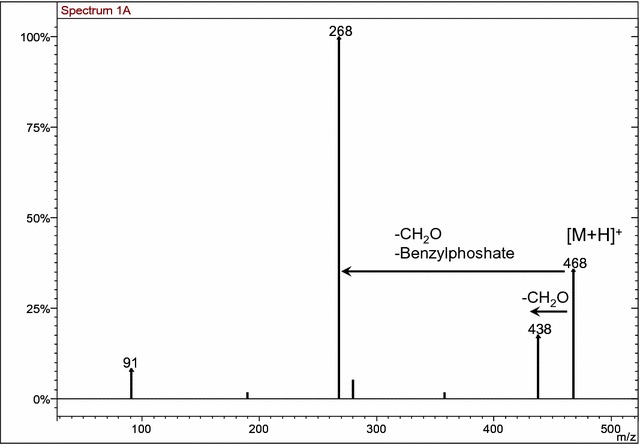
MS/MS spectra of [M+H]^+^ species of compound **10a**

**Fig. 8:**
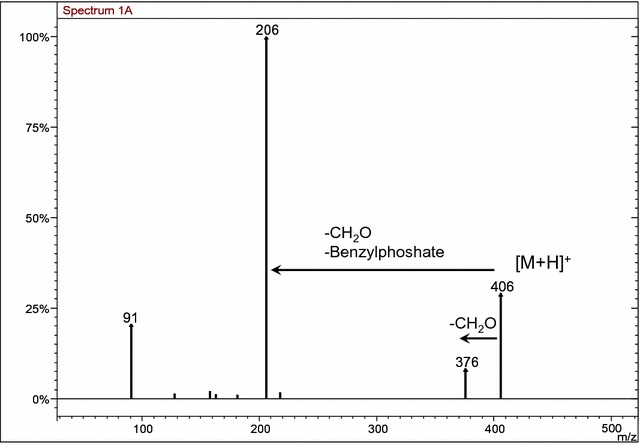
MS/MS spectra of [M+H]^+^ species of compound **10b**

In order to verify this hypothesis, we extended the MS/MS analysis to other derivatives of the thiazolone ring with the position of the dibenzylphosphate group unknown. MS/MS analysis of the [M+H]^+^ species of compounds **10a** and **10b** demonstrated that both analytes lost formaldehyde, with rearrangement of the benzyloxy group (Figs. 7 and 8). This behavior was characteristic of the O-dibenzylphosphate derivative **9** discussed above. Therefore, the structures of compounds **10a** and **10b** were assigned as O-dibenzylphosphate derivatives.

## Results and discussion

**Fig. 9:**
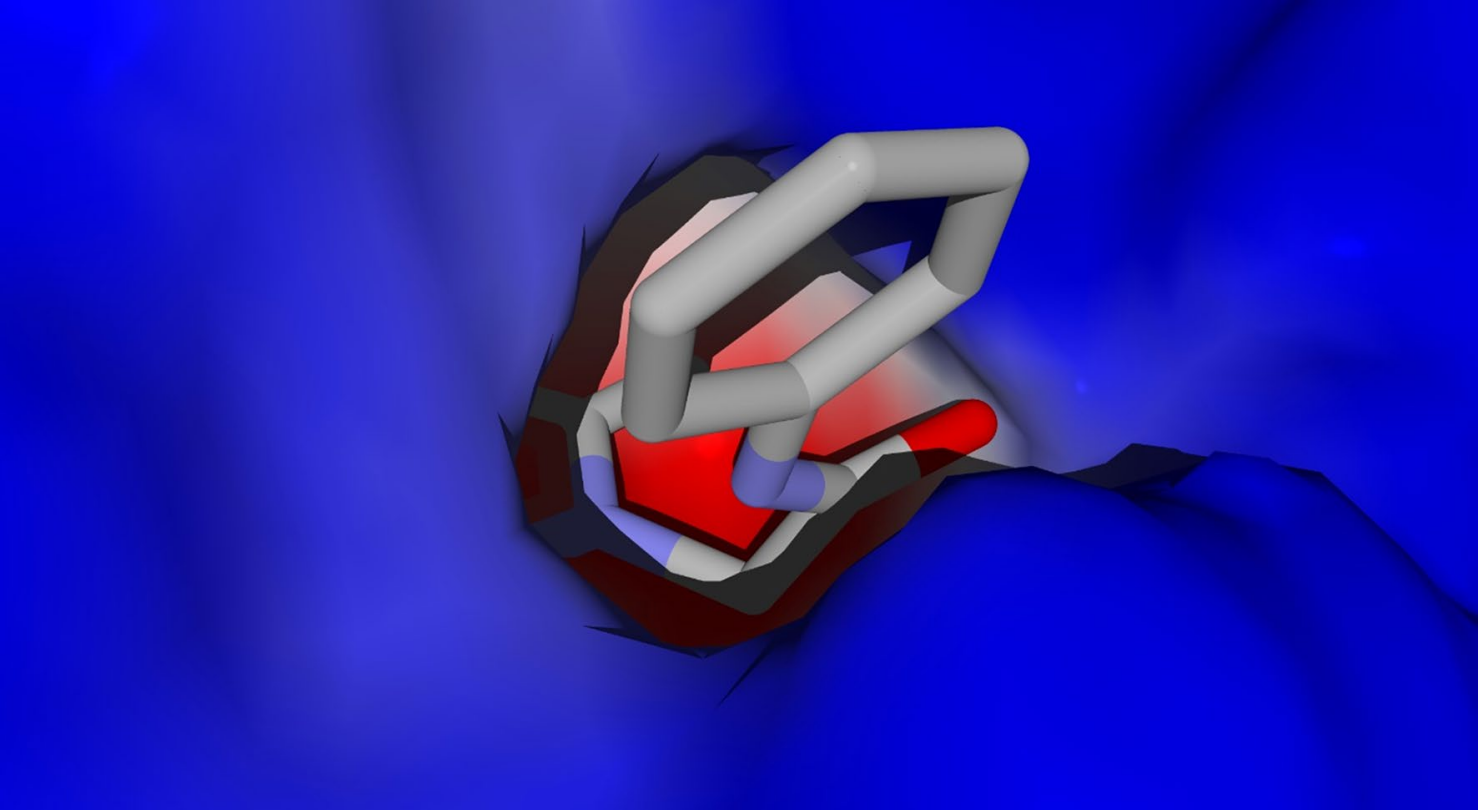
Docking pose of compound **2e** within the HNE ligand-binding site

After the correct assignment of the structures, all new compounds were tested as HNE inhibitors (see “Experimental section”). Unfortunately, no activity was found at the highest concentration tested (40 µM) for all the compounds in the series. To explain the inability of the investigated compounds to inhibit HNE, we have performed molecular docking studies for docking of compound **2e** into the elastase binding site using MVD software. Our approach was analogous to that with we applied in our earlier investigation of HNE inhibitors [16]. Considering conformational flexibility of the ligand and of the side chains of 42 residues, we found an optimum docking pose of compound **2e** into the HNE binding site (Fig. 9). It is known that inhibitory activity towards serine proteases depends on the ability of a compound to form a Michaelis complex with the serine residue at the center of the oxyanion hole and belonging to the catalytic triad Ser…His…Asp [24, 25]. Normally, this complex is formed via nucleophilic attack by the serine oxygen to the carbonyl carbon atom of a ligand and is controlled by geometric peculiarities of the interaction. Namely, the distance O(Ser195)…C(carbonyl in ligand) should be relatively short, and the corresponding angle O(Ser195)…C=O should fall within the interval of 80–120 degrees to support successful formation of the Michaelis complex with HNE [24, 25].

**Fig. 10:**
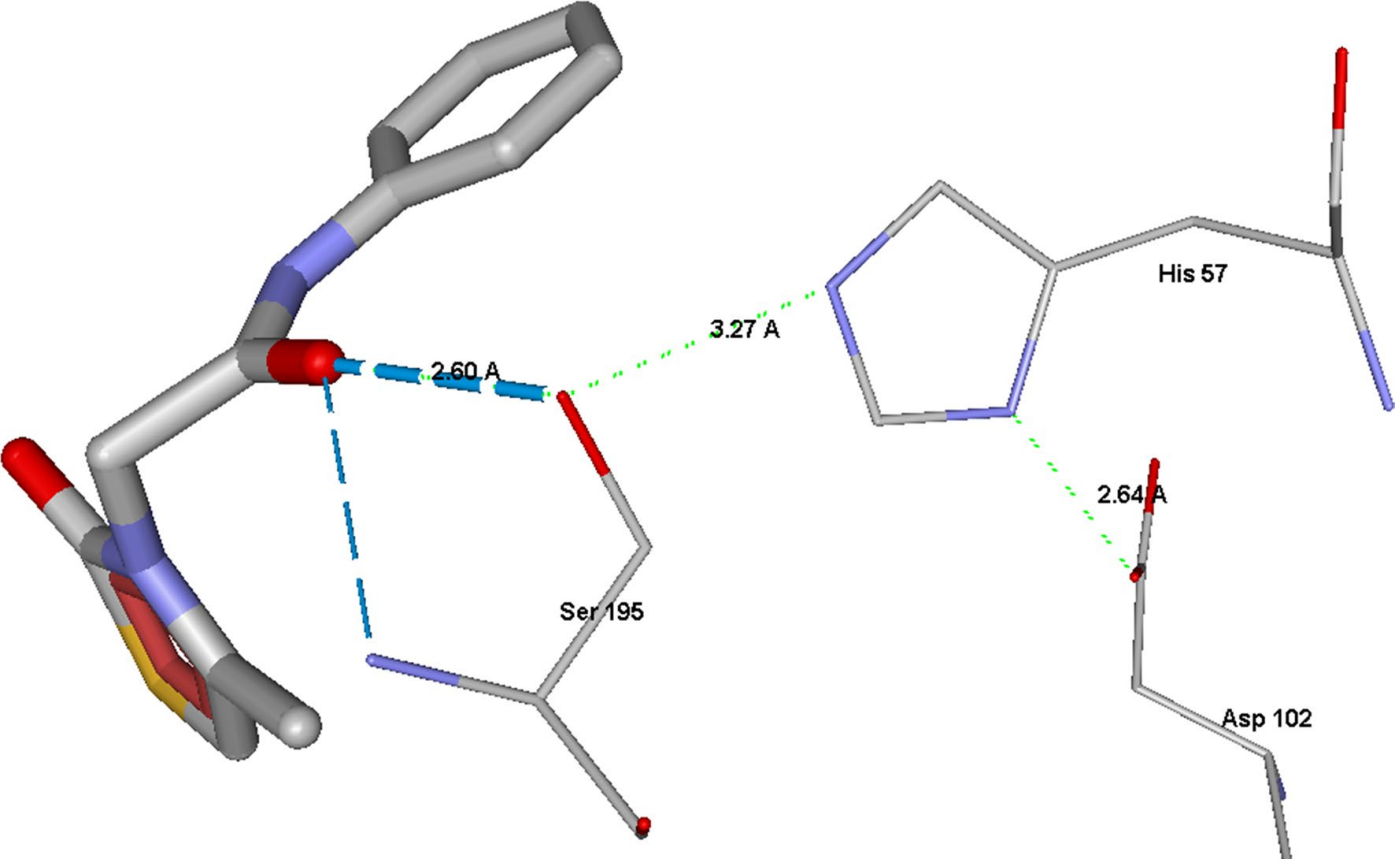
Orientation compound **2e** docking pose (thick cylinders) with respect to residues of the catalytic triad (thin cylinders). Hydrogen atoms are hidden for clarity. Hydrogen bonds are shown by blue dashed lines. Segments of the proton transfer channel are indicated as light-green dashed lines

We found that compound **2e** is strongly H-bonded to Ser195 by its oxygen atom, and the distance O(Ser195)…C(carbonyl in ligand) is d_1_ = 3.77 Å. In addition, the inter-residue distances between heteroatoms in the catalytic triad Ser195…His57…Asp102 were 3.27 and 2.64 Å, respectively (Fig. 10). The sum of the latter two distances gives the length of the proton transfer channel L = 5.91 Å. Relatively low values of d_1_ and L are favorable for Michaelis complex formation. However, the low-energy pose of molecule **2e** forms an angle O(Ser195)…C=O of 12°. This angle is too acute and makes the nucleophilic attack on the carbonyl group impossible with such an orientation of the ligand. It should be noted that the pose is fixed in a narrow binding site cavity (Fig. 9), hence the molecule cannot easily change its orientation to satisfy the conditions necessary for the Michaelis complex formation.

## Conclusion

Overall, the thiazol-2-(3*H*)-one nucleus is not a good scaffold for HNE inhibitors, as the endocyclic carbonyl group does not represent an ideal point of attack for catalytic Ser195 in HNE. Despite the negative result, we obtained some important structure–function information from this study. In general, both the docking and low-energy pose results obtained for compound **2e** explain the lack of inhibitory activity for this library of thiazol-2-(3*H*)-one-based compounds, as compared to the reference series of isoxazolones [18]. Additionally, compounds **3a**–**f**, **5c**, and **6c**, which have only a carbonyl group at position 2 of the thiazol-2-(3*H*)-one nucleus, were inactive, confirming the absence of Ser195 nucleophilic attack on the endocyclic carbonyl function of this series. On the other hand, the inactivity of compounds **2a**–**f**, **5a**, **b**, and **6a**, **b**, bearing a different carbonyl group (i.e., amide or ester) at N-1, suggests that the thiazol-2-(3*H*)-one nucleus itself is not a suitable scaffold for HNE inhibitors. The lack of activity for compounds **8**–**10** is a further support for this conclusion, considering the presence of a bulky phosphonic fragment, which is a typical residue in potent HNE inhibitors reported in the literature [26].

## Experimental section

### Chemistry

All melting points were determined on a Büchi apparatus and are uncorrected. ^1^H NMR, ^13^C-NMR, HSQC and HMBC spectra were recorded on an Avance 400 instrument (Bruker Biospin Version 002 with SGU). Chemical shifts are reported in ppm, using the solvent as internal standard. Extracts were dried over Na_2_SO_4_, and the solvents were removed under reduced pressure. Merck F-254 commercial plates were used for analytical TLC to follow the course of the reactions. Silica gel 60 (Merck 70–230 mesh) was used for column chromatography. Microanalyses were performed with a Perkin-Elmer 260 elemental analyzer for C, H, and N, and the results were within ± 0.4% of the theoretical values unless otherwise stated. Reagents and starting material were commercially available.

### General procedure for 2a–f

1.12 mmol of K_2_CO_3_ and 0.67 mmol (for **2a**–**f**) of the appropriate 2-chloro-*N*-phenylacetamide were added to a suspension of intermediate **1a**–**c** [21–23] (0.56 mmol) in anhydrous CH_3_CN (3 mL). The mixture was stirred at reflux for 6–7 h. After evaporation of the solvent, ice-cold water (20 mL) was added, and the precipitate was recovered by vacuum filtration. Final compounds **2a**–**f** were purified by column chromatography using cyclohexane/ethyl acetate 2:1 (**2a**, **b**, **e**), 1:2 (**2c**, **d**) and 1:1 (**2f**) as eluents.

#### *2*-*(2*-*Oxo*-*4*-*phenylthiazol*-*3(2H)*-*yl)*-*N*-*phenylacetamide (****2a****)*

Yield = 34%; mp = 177–179 °C (EtOH). ^1^H-NMR (CDCl_3_) δ 4.45 (s, 2H, CH_2_), 6.14 (s, 1H, CH), 7.07 (t, 1H, Ar, *J* = 7.2 Hz), 7.25 (t, 2H, Ar, *J* = 7.6 Hz), 7.40–7.50 (m, 7H, Ar), 8.63 (exch br s, 1H, NH). ^13^C-NMR (CDCl3) δ 53.40 (CH_2_), 110.53 (CH), 121.61 (CH), 121.65 (CH), 127.90 (CH), 128.05 (CH), 128.32 (CH), 128.36 (CH), 128.63 (CH), 128.68 (CH), 128.91 (CH), 128.96 (CH), 130.70 (C), 131.92 (C), 138.50 (C), 168.54 (C), 169.93 (C). IR: 1599 cm^−1^ (C=O), 1659 cm^−1^ (C=O), 3262 cm^−1^ (NH). LC–MS: 311.0 [M+H]^+^.

#### *N*-*(2,6*-*dichlorophenyl)*-*2*-*(2*-*oxo*-*4*-*phenylthiazol*-*3(2H)*-*yl)acetamide (****2b****)*

Yield = 10%; mp = 213–216 °C (EtOH). ^1^H-NMR (CDCl_3_) δ 4.51 (s, 2H, CH_2_), 6.18 (s, 1H, CH), 7.21 (t, 1H, Ar, *J* = 8.0 Hz), 7.39 (d, 2H, Ar, *J* = 8.4 Hz), 7.45–7.55 (m, 5H, Ar), 8.22 (exch br s, 1H, NH). ^13^C-NMR (CDCl3) δ 53.41 (CH_2_), 110.50 (CH), 126.11 (CH), 127.90 (CH), 128.24 (CH), 128.27 (CH), 128.33 (CH), 128.36 (CH), 128.63 (CH), 128.69 (CH), 130.70 (C), 131.94 (C), 132.06 (C), 133.30 (C), 133.35 (C) 168.50 (C), 169.96 (C). IR: 1585 cm^−1^ (C=O), 1646 cm^−1^ (C=O), 3205 cm^−1^ (NH). LC–MS: 379.9 [M+H]^+^.

#### *2*-*(5*-*Acetyl*-*4*-*methyl*-*2*-*oxothiazol*-*3(2H)*-*yl)*-*N*-*phenylacetamide (****2c****)*

Yield = 16%; mp = 188–190 °C (EtOH). ^1^H-NMR (CDCl_3_) δ 2.40 (s, 3H, CH_3_), 2.68 (s, 3H, COCH_3_), 4.59 (s, 2H, CH_2_), 7.15 (t, 1H, Ar, *J* = 7.4 Hz), 7.33 (t, 2H, Ar, *J* = 7.6 Hz), 7.49 (d, 2H, Ar, *J* = 8.0 Hz), 8.10 (exch br s, 1H, NH). ^13^C-NMR (CDCl_3_) δ 14.70 (CH_3_), 26.14 (CH_3_), 53.52 (CH_2_), 107.41 (C), 121.60 (CH), 121.66 (CH), 128.04 (CH), 128.92 (CH), 128.97 (CH), 138.55 (C), 145.68 (C), 168.53 (C), 169.91 (C), 192.70 (C). IR: 1575 cm^−1^ (C=O), 1643 cm^−1^ (C=O), 1698 cm^−1^ (C=O), 3295 cm^−1^ (NH). LC–MS: 291.1 [M+H]^+^.

#### *2*-*(5*-*Acetyl*-*4*-*methyl*-*2*-*oxothiazol*-*3(2H)*-*yl)*-*N*-*(2,6*-*dichlorophenyl)acetamide (****2d****)*

Yield = 15%; mp = 209–211 °C (EtOH). ^1^H-NMR (CDCl_3_) δ 2.40 (s, 3H, CH_3_), 2.68 (s, 3H, COCH_3_), 4.69 (s, 2H, CH_2_), 7.21 (t, 1H, Ar, *J* = 8.0 Hz), 7.37 (d, 2H, Ar, *J* = 8.4 Hz), 7.82 (exch br s, 1H, NH). ^13^C-NMR (CDCl3) δ 14.74 (CH_3_), 26.11 (CH_3_), 53.56 (CH_2_), 107.40 (C), 126.13 (CH), 128.22 (CH), 128.26 (CH), 132.05 (C), 133.30 (C), 133.33 (C), 145.68 (C), 168.52 (C), 169.93 (C), 192.71 (C). IR: 1573 cm^−1^ (C=O); 1637 cm^−1^ (C=O), 1672 cm^−1^ (C=O), 3202 cm^−1^ (NH). LC–MS: 359.9 [M+H]^+^.

#### *2*-*(4*-*Methyl*-*2*-*oxothiazol*-*3(2H)*-*yl)*-*N*-*phenylacetamide (****2e****)*

Yield = 26%; mp = 188–191 °C (EtOH). ^1^H-NMR (CDCl_3_) δ 2.26 (s, 3H, CH_3_), 4.51 (s, 2H, CH_2_), 5.86 (s, 1H, CH), 7.13 (t, 1H, Ar, *J* = 7.4 Hz), 7.32 (t, 2H, Ar, *J* = 7.6 Hz), 7.50 (d, 2H, Ar, *J* = 8.0 Hz), 8.39 (exch br s, 1H, NH). ^13^C-NMR (CDCl3) δ 17.32 (CH_3_), 53.51 (CH_2_), 101.90 (CH), 120.45 (C), 121.63 (CH), 121.67 (CH), 128.04 (CH), 128.93 (CH), 128. 98 (CH), 138.50 (C), 168.58 (C), 169.92 (C). IR: 1633 cm^−1^ (C=O), 1692 cm^−1^ (C=O), 3274 cm^−1^ (NH). LC–MS: 249.1 [M+H]^+^.

#### *N*-*(2,6*-*dichlorophenyl)*-*2*-*(4*-*methyl*-*2*-*oxothiazol*-*3(2H)*-*yl)acetamide (****2f****)*

Yield = 9%; mp = 266–269 °C (EtOH). ^1^H-NMR (CDCl_3_) δ 2.28 (s, 3H, CH_3_), 4.61 (s, 2H, CH_2_), 5.89 (s, 1H, CH), 7.20 (t, 1H, Ar, *J* = 8.0 Hz), 7.37 (d, 2H, Ar, *J* = 8.0 Hz), 7.90 (exch br s, 1H, NH). ^13^C-NMR (CDCl3) δ 17.30 (CH_3_), 53.52 (CH_2_), 101.90 (CH), 120.48 (C), 126.11 (CH), 128.24 (CH), 128. 29 (CH), 132.01 (C), 133.33 (C), 133.38 (C), 168.50 (C), 169.91 (C). IR: 1634 cm^−1^ (C=O), 1685 cm^−1^ (C=O), 3197 cm^−1^ (NH). LC–MS: 317.9 [M+H]^+^.

### General procedure for 3a–f

A mixture of intermediate of type **1** (**1a**–**c**) [21–23] (0.62 mmol) the appropriate phenylboronic acid (1.24 mmol), Cu(Ac)_2_ (0.93 m mol), and Et_3_N (1.24 mmol) in dry CH_2_Cl_2_ (3 mL), was stirred at room temperature overnight. The organic layer was washed with water (3 × 15 mL) and then with 33% aqueous ammonia (3 × 10 mL). The organic layer was dried over sodium sulfate and the solvent was evaporated in vacuo to obtain final compounds **3a**–**f**, which were purified by column chromatography using cyclohexane/ethyl acetate 5:1 (for **3a**), 2:1 (for **3b**) or 3:1 (for **3c**–**f**) as eluents.

#### *4*-*Phenyl*-*3*-*m*-*tolyl*-*thiazol*-*2(3H)*-*one (****3a****)*

Yield = 12%; mp = 118–121 °C (EtOH). ^1^H-NMR (CDCl_3_) δ 2.31 (s, 3H, CH_3_), 6.20 (s, 1H, CH), 6.88 (d, 1H, Ar, *J* = 7.6 Hz), 7.02 (s, 1H, Ar), 7.09–7.15 (m, 2H, Ar), 7.18–7.26 (m, 5H, Ar). ^13^C-NMR (CDCl3) δ 21.30 (CH_3_), 106.33 (CH), 124.67 (CH), 125.11 (CH), 127.92 (CH), 128.30 (CH), 128.35 (CH), 128.63 (CH), 128.68 (CH), 128.88 (CH), 129.55 (CH), 130.41 (C), 132.64 (C), 138.62 (C), 147.36 (C), 166.70 (C). IR: 1658 cm^−1^ (C=O). LC–MS: 268.1 [M+H]^+^.

#### *4*-*Phenyl*-*3*-*[3*-*(trifluoromethyl)phenyl]thiazol*-*2(3H)*-*one (****3b****)*

Yield = 15%; mp = 84–87 °C (EtOH). ^1^H-NMR (CDCl_3_) δ 6.26 (s, 1H, CH), 7.06 (d, 2H, Ar, *J* = 7.6 Hz), 7.24–7.30 (m, 3H, Ar), 7.35–7.40 (m, 2H, Ar), 7.47 (t, 1H, Ar, *J* = 7.8 Hz), 7.55 (d, 1H, Ar, *J* = 7.6 Hz). ^13^C-NMR (CDCl3) δ 106.30 (CH), 120.72 (CH), 124.11 (C), 125.93 (CH), 127.97 (CH), 128.35 (CH), 128.39 (CH), 128.65 (CH), 128.68 (CH), 129.25 (CH), 130.41 (C), 131.24 (C), 131.40 (CH), 133.06 (C), 147.35 (C), 166.71 (C). IR: 1656 cm^−1^ (C=O). LC–MS: 322.0 [M+H]^+^.

#### *5*-*Acetyl*-*4*-*methyl*-*3*-*m*-*tolylthiazol*-*2(3H)*-*one (****3c****)*

Yield = 12%; mp = 104–106 °C (EtOH). ^1^H-NMR (CDCl_3_) δ 2.31 (s, 3H, CH_3_), 2.42 (s, 3H, COCH_3_), 2.43 (s, 3H, CH_3_-Ph), 7.04–7.09 (m, 2H, Ar), 7.32 (d, 1H, Ar, *J* = 7.6 Hz), 7.42 (t, 1H, Ar, J = 7.6 Hz). ^13^C-NMR (CDCl3) δ 15.03 (CH_3_), 21.33 (CH_3_), 26.12 (CH_3_), 103.27 (C), 124.60 (CH), 125.14 (CH), 128.88 (CH), 129.55 (CH), 132.60 (C), 138.62 (C), 154.03 (C), 166.74 (C), 192.71 (C). IR: 1629 cm^−1^ (C=O), 1670 cm^−1^ (C=O). LC–MS: 248.1 [M+H]^+^.

#### *5*-*Acetyl*-*4*-*methyl*-*3*-*[3*-*(trifluoromethyl)phenyl]thiazol*-*2(3H)*-*one (****3d****)*

Yield = 11%; mp = 147–150 °C (EtOH). ^1^H-NMR (CDCl_3_) δ 2.33 (s, 3H, CH_3_), 2.43 (s, 3H, COCH_3_), 7.48 (d, 1H, Ar, *J* = 8.0 Hz), 7.53 (s, 1H, Ar), 7.71 (t, 1H, Ar, *J* = 7.8 Hz), 7.80 (d, 1H, Ar, J = 8.0 Hz). ^13^C-NMR (CDCl3) δ 15.05 (CH_3_), 26.14 (CH_3_), 103.25 (C), 120.70 (CH), 124.13 (C), 125.97 (CH), 129.24 (CH), 131.22 (C), 131.40 (CH), 133.04 (C), 154.08 (C), 166.77 (C), 192.75 (C). IR: 1637 cm^−1^ (C=O), 1681 cm^−1^ (C=O). LC–MS: 302.0 [M+H]^+^.

#### *4*-*Methyl*-*3*-*m*-*tolyl*-*thiazol*-*2(3H)*-*one (****3e****)*

Yield = 11%; mp = 69–72 °C (EtOH). ^1^H-NMR (CDCl_3_) δ 1.90 (s, 3H, CH_3_), 2.42 (s, 3H, CH_3_-Ph), 5.86 (s, 1H, CH), 7.05–7.10 (m, 2H, Ar), 7.25 (s, 1H, Ar), 7.39 (t, 1H, Ar, *J* = 7.6 Hz). ^13^C-NMR (CDCl3) δ 21.32 (CH_3_), 21.79 (CH_3_), 97.70 (CH), 124.65 (CH), 125.16 (CH), 128.83 (CH), 129.51 (CH), 132.64 (C), 138.60 (C), 149.33 (C), 166.75 (C). IR: 1640 cm^−1^ (C=O). LC–MS: 206.0 [M+H]^+^.

#### *4*-*Methyl*-*3*-*[3*-*(trifluoromethyl)phenyl]thiazol*-*2(3H)*-*one (****3f****)*

Yield = 18%; Oil. ^1^H-NMR (CDCl_3_) δ 1.92 (s, 3H, CH_3_), 5.92 (s, 1H, CH), 7.50 (d, 1H, Ar, *J* = 7.6 Hz), 7.56 (s, 1H, Ar), 7.66 (t, 1H, Ar, *J* = 7.8 Hz), 7.73 (d, 1H, Ar, *J* = 7.6 Hz). ^13^C-NMR (CDCl_3_) δ 15.92 (CH_3_), 96.97 (CH_2_), 122.05 (C), 124.75 (C), 125.37 (CH), 125.90 (CH), 130.32 (CH), 131.88 (CH), 132.04 (C), 136.19 (C), 172.47 (C). IR: 1645 cm^−1^ (C=O). LC–MS: 260.0 [M+H]^+^.

### General procedure for 5a–c and 6a–c

To a suspension of the substrate of type **1** (**1a**, **b**) [21, 23] (0.54 mmol) in anhydrous CH_3_CN (3 mL), 1.08 mmol of K_2_CO_3_ and 0.65 mmol of the appropriate commercially available intermediate **4a**, **b** were added. The mixture was stirred at reflux for 8 h (**5a**, **b** and **6a**, **b**) or for 3 h (**5c-** and **6c**). After evaporation of the solvent, ice-cold water (20 mL) was added, and the suspension was recovered by extraction with ethyl acetate (3 × 15 mL). The organic layer was dried over sodium sulfate, and the solvent was evaporated in vacuo to obtain final compounds **5a**–**c** and **6a**–**c**, which were purified by column chromatography using cyclohexane/ethyl acetate 3:1 (for **5a** and **6a**), 2:1 (for **5b** and **6b**) or 5:1 (for **5c** and -**6c**) as eluents.

#### *(2*-*Oxo*-*4*-*phenylthiazol*-*3(2H)*-*yl)methyl benzoate (****5a****)*

Yield = 39%; Oil. ^1^H-NMR (CDCl_3_) δ 5.85 (s, 2H, CH_2_), 6.12 (s, 1H, CH), 7.42–7.48 (m, 7H, Ar), 7.61 (t, 1H, Ar, *J* = 7.6 Hz), 8.01 (d, 2H, Ar, *J* = 8.0 Hz). ^13^C-NMR (CDCl_3_) δ 67.08 (CH_2_), 99.45 (CH), 128.13 (CH), 128.26 (CH), 128.50 (CH), 128.85 (CH), 129.04 (CH), 130.13 (C), 130.19 (CH), 130.48 (CH), 136.92 (C), 165.11 (C), 172.78 (C). IR: 1681 cm^−1^ (C=O), 1724 cm^−1^ (C=O). LC–MS: 312.1 [M+H]^+^.

#### *(5*-*Acetyl*-*4*-*methyl*-*2*-*oxothiazol*-*3(2H)*-*yl)methyl benzoate (****5b****)*

Yield = 21%; mp = 121–123 °C (EtOH). ^1^H-NMR (CDCl_3_) δ 2.39 (s, 3H, CH_3_), 2.69 (s, 3H, COCH_3_), 6.03 (s, 2H, CH_2_), 7.46 (t, 2H, Ar, *J* = 7.8 Hz), 7.62 (t, 1H, Ar, *J* = 7.6 Hz), 8.03 (d, 2H, Ar, *J* = 8.4 Hz). ^13^C-NMR (CDCl_3_) δ 13.36 (CH_3_), 30.33 (CH_3_), 65.45 (CH_2_), 98.49 (CH), 113.43 (C), 128.15 (CH), 128.50 (CH), 128.61 (CH), 129.92 (CH), 133.89 (CH), 141.15 (C), 165.10 (C), 169.90 (C), 189.90 (C). IR: 1643 cm^−1^ (C=O), 1698 cm^−1^ (C=O), 1720 cm^−1^ (C=O). LC–MS: 292.0 [M+H]^+^.

#### *3*-*(3*-*Methylbenzyl)*-*4*-*phenylthiazol*-*2(3H)*-*one (****5c****)*

Yield = 13%; Oil. ^1^H-NMR (CDCl_3_) δ 2.72 (s, 3H, CH_3_), 4.87 (s, 2H, CH_2_), 6.03 (s, 1H, CH), 6.72–6.78 (m, 2H, Ar), 7.03 (d, 1H, Ar, *J* = 7.6 Hz), 7.12 (t, 1H, Ar, *J* = 7.4 Hz), 7.19 (d, 2H, Ar, *J* = 7.0 Hz), 7.30–7.40 (m, 3H, Ar). ^13^C-NMR (CDCl_3_) δ 21.65 (CH_3_), 51.03 (CH_2_), 110.54 (CH), 123.97 (CH), 127.02 (CH), 127.96 (CH), 128.31 (CH), 128.37 (CH), 128.40 (CH), 128.63 (CH), 128.66 (CH), 128.89 (CH), 130.70 (C), 131.92 (C), 138.26 (C), 138.33 (C), 169.90 (C). IR: 1661 cm^−1^ (C=O). LC–MS: 282.1 [M+H]^+^.

#### *(4*-*Phenylthiazol*-*2*-*yloxy)methyl benzoate (****6a****)*

Yield = 28%; Oil. ^1^H-NMR (CDCl_3_) δ 6.41 (s, 2H, CH_2_), 6.97 (s, 1H, CH), 7.34 (d, 1H, Ar, *J* = 7.8 Hz), 7.36–7.44 (m, 4H, Ar), 7.47 (t, 1H, Ar, *J* = 7.8 Hz), 7.86 (d, 2H, Ar, *J* = 7.8 Hz), 8.13 (d, 2H, Ar, *J* = 8.0 Hz). ^13^C-NMR (CDCl_3_) δ 85.48 (CH_2_), 105.95 (CH), 125.94 (CH), 128.06 (CH), 128.55 (CH), 128.68 (CH), 129.18 (CH), 130.14 (CH), 133.74 (CH), 134.31 (C), 149.26 (C), 165.26 (C), 171.56 (C). IR: 1737 cm^−1^ (C=O). LC–MS: 312.1 [M+H]^+^.

#### *(5*-*Acetyl*-*4*-*methylthiazol*-*2*-*yloxy)methyl benzoate (****6b****)*

Yield = 10%; Oil. ^1^H-NMR (CDCl_3_) δ 2.47 (s, 3H, CH_3_), 2.64 (s, 3H, COCH_3_), 6.31 (s, 2H, CH_2_), 7.49 (t, 2H, Ar, *J* = 7.8 Hz), 7.63 (t, 1H, Ar, *J* = 7.4 Hz), 8.11 (d, 2H, Ar, *J* = 8.4 Hz). ^13^C-NMR (CDCl_3_) δ 16.86 (CH_3_), 26.83 (CH_3_), 94.35 (CH_2_), 128.60 (CH), 128.63 (CH), 129.95 (CH), 129.99 (CH), 130.10 (C), 133.04 (CH), 143.05 (C), 153.01 (C), 161.07 (C), 165.90 (C), 196.96 (C). IR: 1695 cm^−1^ (C=O), 1735 cm^−1^ (C=O). LC–MS: 292.0 [M+H]^+^.

#### *2*-*(3*-*Methylbenzyloxy)*-*4*-*phenylthiazole (****6c****)*

Yield = 13%; Oil. ^1^H-NMR (CDCl_3_) δ 2.42 (s, 3H, CH_3_), 5.53 (s, 2H, CH_2_), 6.90 (s, 1H, CH), 7.20–7.25 (m, 1H, Ar), 7.30–7.35 (m, 4H, Ar), 7.40–7.45 (m, 2H, Ar), 7.88 (d, 2H, Ar, *J* = 8.0 Hz). ^13^C-NMR (CDCl_3_) δ 21.65 (CH_3_), 70.83 (CH_2_), 110.84 (CH), 124.17 (CH), 127.52 (CH), 127.56 (CH), 127.91 (CH), 128.77 (CH), 128.80 (CH), 129.03 (CH), 129.26 (CH), 129.29 (CH), 133.07 (C), 138.62 (C), 141.16 (C), 152.33 (C), 154.05 (C). LC–MS: 282.1 [M+H]^+^.

### General procedure for 8, 9 and 10a, b

1.02 mmol of K_2_CO_3_ and 0.61 mmol of dibenzyl chloromethyl phosphate (**7**) [27] were added to a suspension of the substrate **1a**–**c** [21–23] (0.51 mmol) in anhydrous CH_3_CN (4 mL). The mixture was stirred at reflux for 6 h. After evaporation of the solvent, ice-cold water (20 mL) was added and the suspension was recovered by extraction with CH_2_Cl_2_ (3 × 15 mL). The organic layer was dried on sodium sulfate and evaporated in vacuo to obtain the final compounds, which were purified by column chromatography using cyclohexane/ethyl acetate 1:2 (**8**, **9**), 3:1 (**10a**) or hexane/ethyl acetate 3:1 for **10b**, as eluents.

#### *(5*-*Acetyl*-*4*-*methyl*-*2*-*oxothiazol*-*3(2H)*-*yl)methyl dibenzyl phosphate (****8****)*

Yield = 12%; Oil. ^1^H-NMR (CDCl_3_) δ 2.35 (s, 3H, CH_3_), 2.52 (s, 3H, COCH_3_), 5.06 (m, 4H, 2 × CH_2_Ph), 5.55 (d, 2H, CH_2_Cl, *J* = 8.8 Hz), 7.30–7.40 (m, 10H, Ar). ^13^C-NMR (CDCl_3_) δ 13.11 (CH_3_), 30.33 (CH_3_), 67.17 (CH_2_), 70.09 (CH_2_), 113.59 (C), 128.20 (CH), 128.69 (CH), 128.85 (CH), 135.18 (C), 140.88 (C), 169.77 (C), 189.75 (C). IR: 1670 cm^−1^ (C=O), 1701 cm^−1^ (C=O). LC–MS: 448.0 [M+H]^+^.

#### *[(5*-*Acetyl*-*4*-*methylthiazol*-*2*-*yloxy)methyl] dibenzyl phosphate (****9****)*

Yield = 13%; Oil. ^1^H-NMR (CDCl_3_) δ 2.45 (s, 3H, CH_3_), 2.58 (s, 3H, COCH_3_), 5.09 (d, 4H, 2 × CH_2_Ph, *J* = 8.0 Hz), 5.92 (d, 2H, CH_2_Cl, *J* = 14.4 Hz), 7.30–7.40 (m, 10H, Ar). ^13^C-NMR (CDCl_3_) δ 18.47 (CH_3_), 30.13 (CH_3_), 69.78 (CH_2_), 88.12 (CH_2_), 127.93 (CH), 128.62 (CH), 128.70 (CH), 129.21 (CH), 135.30 (C), 154.47 (C), 190.15 (C). IR: 1653 cm^−1^ (C=O). LC–MS: 448.0 [M+H]^+^.

#### *Dibenzyl [(4*-*phenylthiazol*-*2*-*yloxy)methyl] phosphate (****10a****)*

Yield = 18%; Oil. ^1^H-NMR (CDCl_3_) δ 5.10 (d, 4H, 2 × CH_2_Ph, *J* = 8.0 Hz), 6.04 (d, 2H, CH_2_Cl, *J* = 13.6 Hz), 6.96 (s, 1H, CH), 7.31–7.43 (m, 13H, Ar), 7.84 (d, 2H, Ar, *J* = 7.6 Hz). ^13^C-NMR (CDCl_3_) δ 69.71 (CH_2_), 88.50 (CH_2_), 106.13 (CH), 125.95 (CH), 127.94 (CH), 128.11 (CH), 128.59 (CH), 128.66 (CH), 134.14 (C), 135.45 (C), 149.29 (C), 171.00 (C). LC–MS: 468.2 [M+H]^+^.

#### *Dibenzyl [(4*-*methylthiazol*-*2*-*yloxy)methyl] phosphate (****10b****)*

Yield = 15%; Oil. ^1^H-NMR (CDCl_3_) δ 2.27 (s, 3H, CH_3_), 5.09 (d, 4H, 2 × CH_2_Ph, *J* = 7.6 Hz), 5.92 (d, 2H, CH_2_Cl, *J* = 14.0 Hz), 6.33 (s, 1H, CH), 7.30–7.40 (m, 10H, Ar). ^13^C-NMR (CDCl_3_) δ 17.10 (CH_3_), 69.41 (CH_2_), 93.80 (CH_2_), 114.93 (CH), 127.15 (CH), 127.64 (CH), 128.91 (CH), 135.44 (C), 149.69 (C), 153.01 (C). LC–MS: 406.1 [M+H]^+^.

### HNE inhibition assay

Compounds were dissolved in 100% DMSO at 5 mM stock concentrations. The final concentration of DMSO in the reactions was 1%, and this level of DMSO had no effect on enzyme activity. The HNE inhibition assay was performed in black flat-bottom 96-well microtiter plates. Briefly, a buffer solution containing 200 mM Tris–HCl, pH 7.5, 0.01% bovine serum albumin, and 0.05% Tween-20 and 20 mU/mL of HNE (Calbiochem) was added to wells containing different concentrations of each compound. The reaction was initiated by addition of 25 μM elastase substrate (*N*-methylsuccinyl-Ala–Ala-Pro-Val-7-amino-methyl-coumarin, Calbiochem) in a final reaction volume of 100 μL/well. Kinetic measurements were obtained every 30 s for 10 min at 25 °C using a Fluoroskan Ascent FL fluorescence microplate reader (Thermo Electron, MA) with excitation and emission wavelengths at 355 and 460 nm, respectively.

### Instrumental

The LC–MS/MS analysis was carried out using a Varian 1200L triple quadrupole system (Palo Alto, CA, USA) equipped with two Prostar 210 pumps, a Prostar 410 auto sampler, and an electrospray source (ESI) operating in the positive ion mode. The ion sources and ion optics parameters were optimized and 1 µg/mL working solution of the test compounds was injected via syringe pump at 10 µL/min. Raw data were collected and processed by Varian Workstation Version 6.8 software.

### Standard solutions

Stock solutions of analytes were prepared in acetonitrile at 1.0 mg/mL and stored at 4 °C. Working solutions of each analyte were freshly prepared by diluting stock solutions in acetonitrile up to a concentration of 1.0 µg/mL.

### Tandem mass spectrometry experiments

Compounds **8** and **9** are positional isomers and under ESI–MS conditions showed the production of abundant protonated molecules, detected at the same *m/z* value, and only a few fragment ions at very low relative abundance (less than 2%). Therefore, we utilized specific collisionally activated MS/MS decomposition pathways to assign their structures. Accordingly, a series of MS/MS experiments were performed by applying different approaches, based on the potential of a triple quadrupole system.

Energy resolved tandem mass spectrometry (ERMS) experiments [28] were performed to evaluate the collision activated decomposition pathway of isomers **8** and **9**. The ERMS experiments involve a series of product ion scan spectra acquisitions, which were obtained by increasing the collision energy stepwise in the range from 0 to 20 V. The results obtained were used to study the fragmentation of molecular species from each analyte and build its breakdown curves. In detail, the product ion scan spectra were acquired in the m/z range from 50 to 650, scan time 600 ms, and argon was used as collisional gas. The breakdown curves data were obtained by introducing 1.0 µg/mL of each analyte via syringe pump at 10 µL/min; the protonated molecule was isolated and the abundance of product ions were monitored. The values of the relative intensities of MS/MS spectra used to build the breakdown curves were the mean of 50 scans for each collision energy and the collision gas pressure was critically evaluated in order to achieve valid and reproducible fragmentation patterns on the basis of the results obtained [29].

### Docking analysis

A 3-D model of compound **2e** was built and pre-optimized by Chem3D (version 12.0.2) software (ChemBioOffice 2010 Suite). The model was imported into the Molegro Virtual Docker (MVD) program together with the structure of human neutrophil elastase (1HNE entry of Protein Data Bank), where the enzyme is complexed with peptide chloromethyl ketone inhibitor [30]. All water molecules were deleted from the protein model. The search area for docking poses was defined as a sphere with 10 Å radius centered at the nitrogen atom in the five-membered ring of the co-crystallized peptide. Side chains of 42 residues in the vicinity of the binding site were set flexible during docking, as described previously [16]. The HNE catalytic triad comprised of Ser195, His57, and Asp102 was also among the residues with flexible side chains. Simulation of the receptor flexibility was performed with the standard technique built in the Molegro program (Molegro Virtual Docker. User Manual, 2010). Values of 0.9 and 0.7, respectively, were assigned to the “Tolerance” and “Strength” parameters of the MVD “Side chain Flexibility” wizard. Forty docking runs were performed with full flexibility of ligand **2e** around its rotatable bonds. Geometric parameters of the lowest-energy docking pose were determined with measurement tools of MVD software in order to estimate the ability of compound **2e** to form a Michaelis complex between the hydroxyl group of Ser195 and each carbonyl group of the ligand, according to previously reported methods [24, 25].

## Additional file

**Additional file 1: Table S1.** Elemental analysis.
