# Supplementary material for: Synthesis and analytical characterization of new thiazol-2-(3H)-ones as human neutrophil elastase (HNE) inhibitors
Source: Chem Cent J. 2017 Dec 6;11:127. doi: 10.1186/s13065-017-0358-1 (PMC5718994; doi:10.1186/s13065-017-0358-1)
Supplement: Supplementary file 1 — Additional file 1: Table S1. Elemental analysis. [file 13065_2017_358_MOESM1_ESM.docx]

**ADDITIONAL INFORMATION**

**Synthesis and analytical characterization of new thiazol-2-(3H)-ones as human neutrophil elastase (HNE) inhibitors**

Letizia Crocetti^a^, Gianluca Bartolucci^a^, Agostino Cilibrizzi^b^, Maria Paola Giovannoni^a*^, Gabriella Guerrini^a^, Antonella Iacovone^a^, Marta Menicatti^a^, Igor A. Schepetkin^c^, Andrei I. Khlebnikov^d,e^_,_ Mark T. Quinn^c^, and Claudia Vergelli^a^

*^a^NEUROFARBA, Sezione di Farmaceutica e Nutraceutica, Università degli Studi di Firenze, Via Ugo Schiff 6, 50019 Sesto Fiorentino, Italy.*

*^b^Institute of Pharmaceutical Science, King’s College London, 150 Stamford Street, London SE1 9NH, UK*

*^c^Department of Microbiology and Immunology, Montana State University, Bozeman, MT 59717, USA.*

*^d^Department of Biotechnology and Organic Chemistry, Tomsk Polytechnic University, Tomsk 634050, Russia*

*^e^Scientific Research Institute of Biological Medicine, Altai State University, Barnaul 656049, Russia*

**Table of contents**

1. ^1^H-NMR, ^13^C-NMR, HMBC and HSQC spectra of compounds **2a**, **2f**, **5b**, **10a** and **10b**.

2. ^1^H-NMR, ^13^C-NMR, HMBC and HSQC spectra of isomers **5a/6a** and **8/9** (the red circle show the presence or the absence of distinctive cross-peaks between proton and carbon to assign the OH and NH tautomer form).

3. Elemental analysis (Table S1).


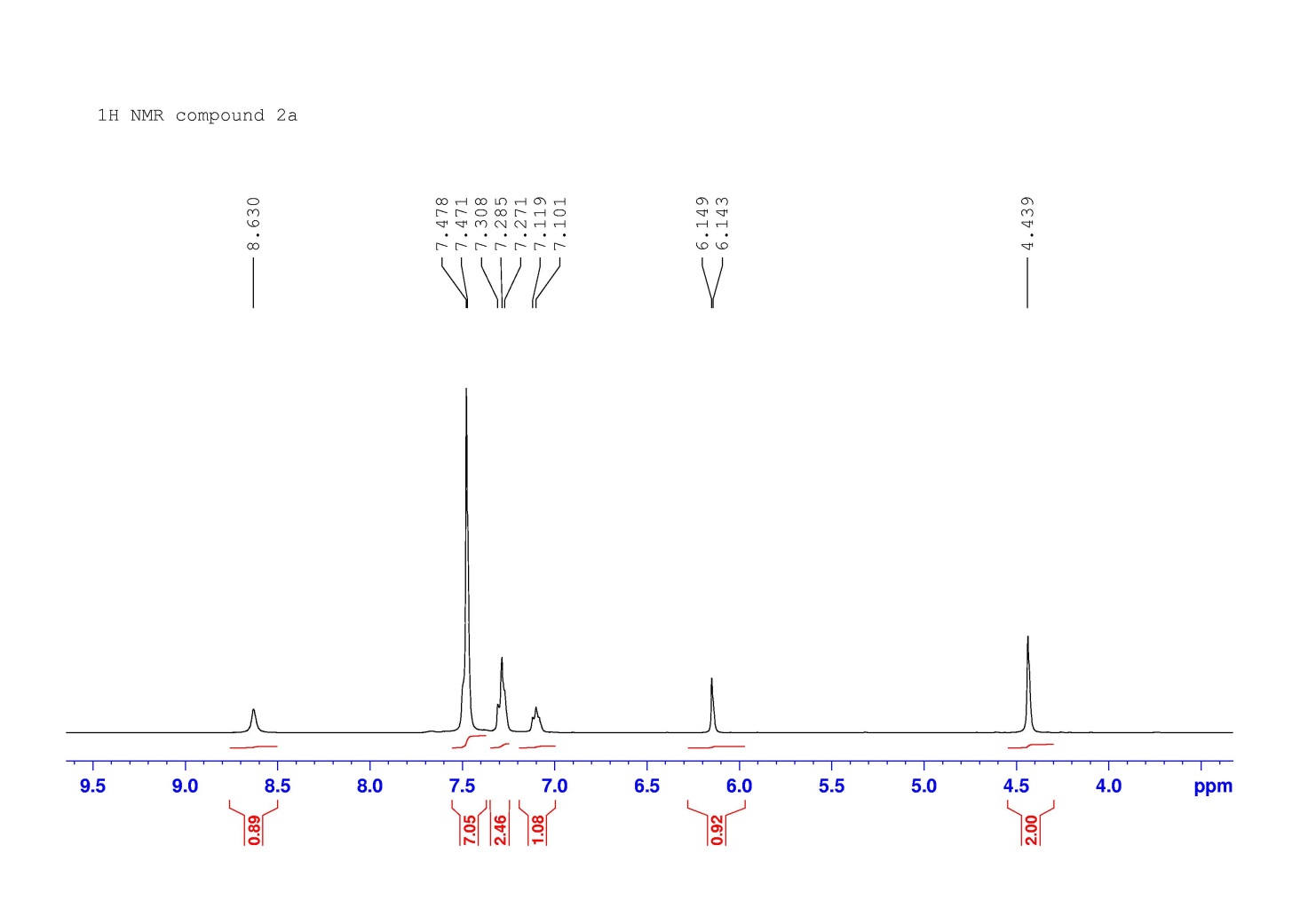

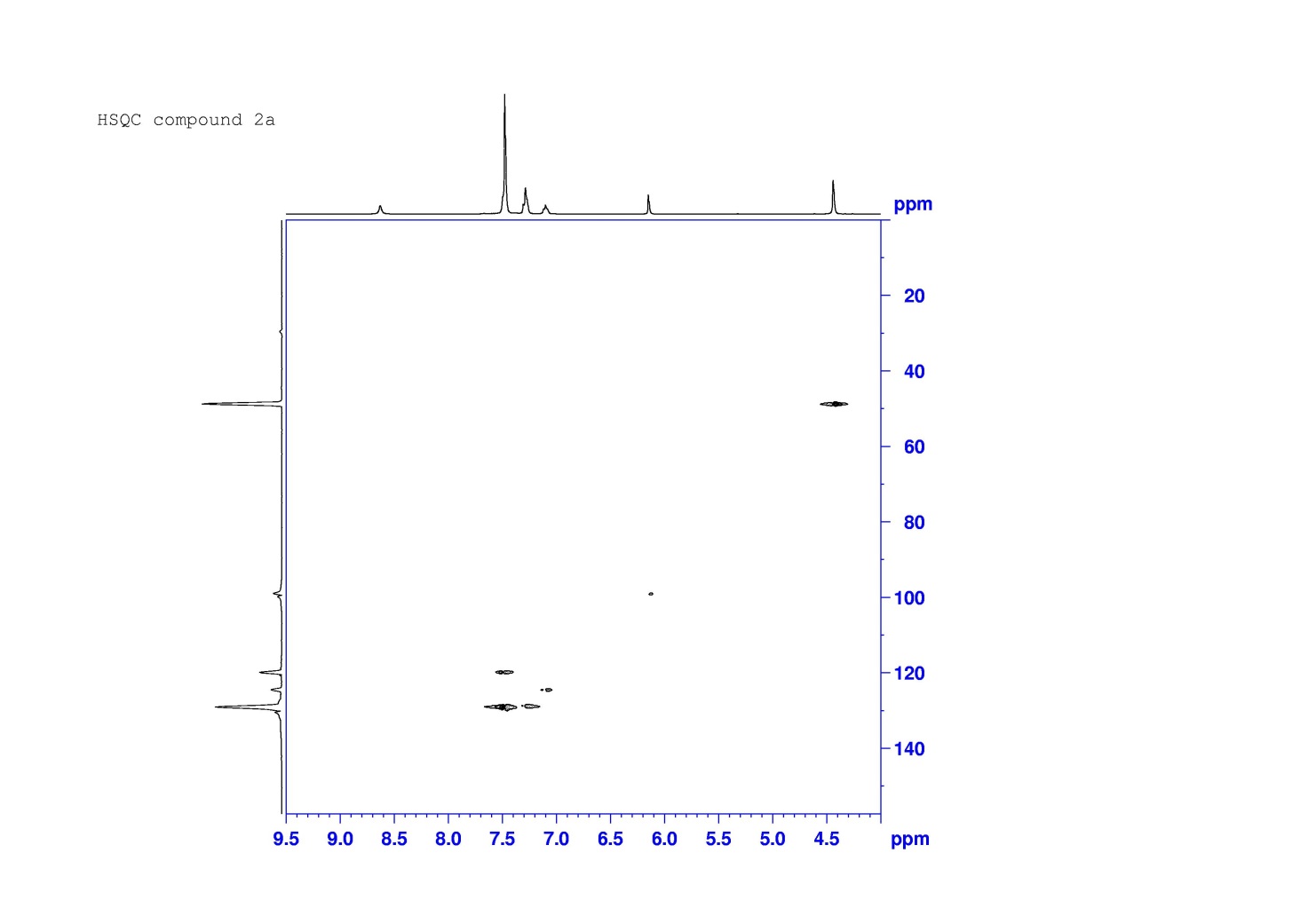


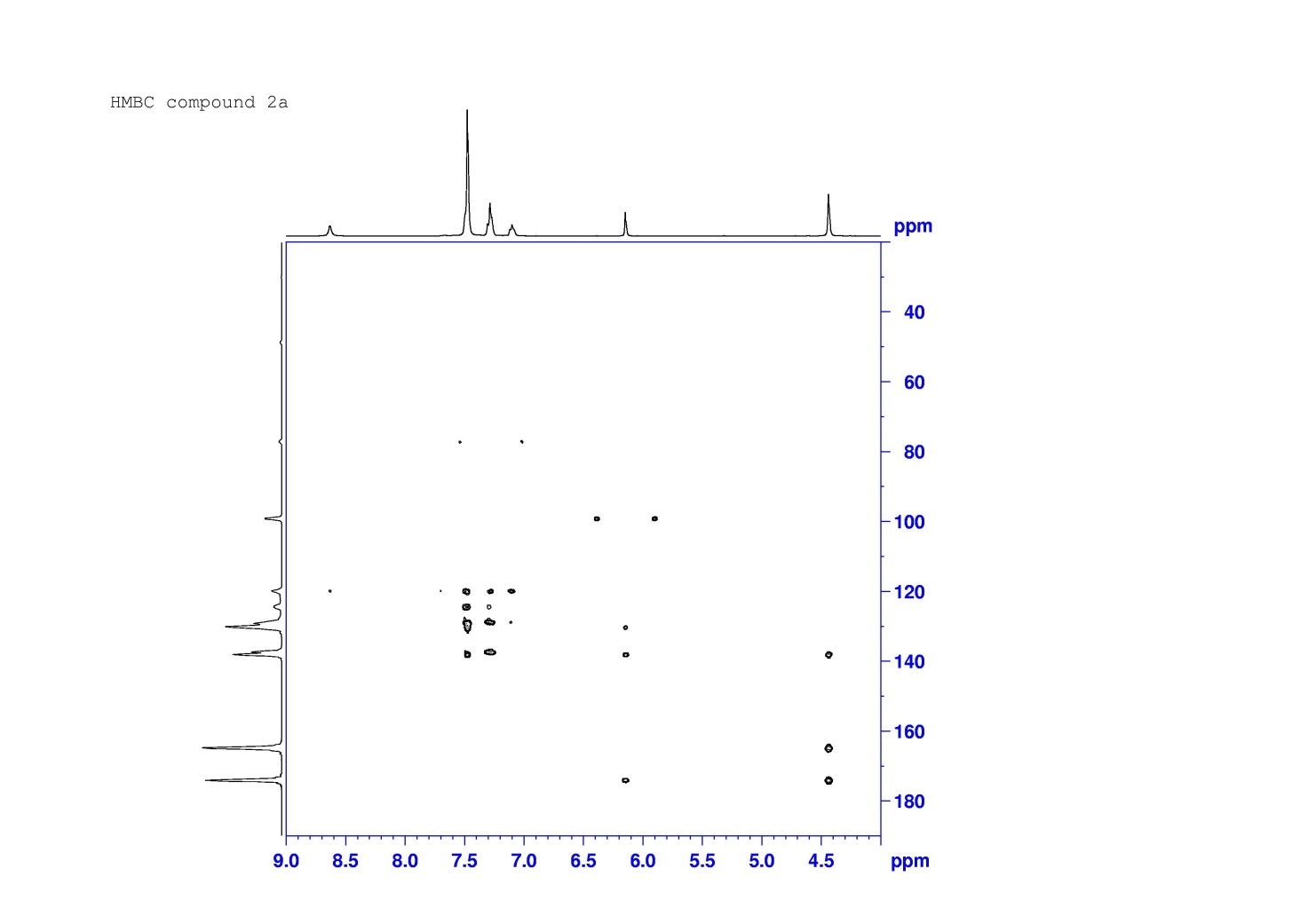

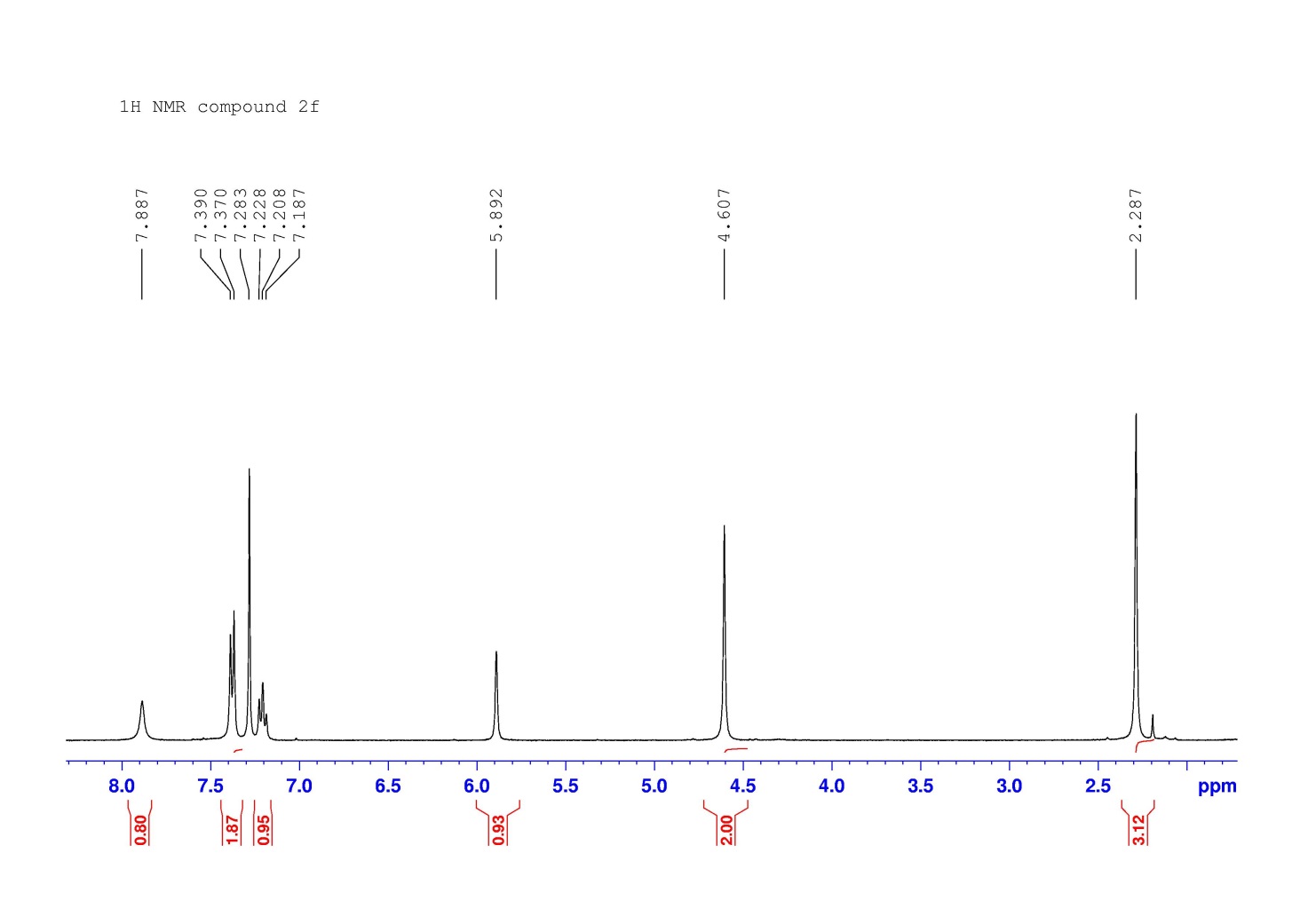


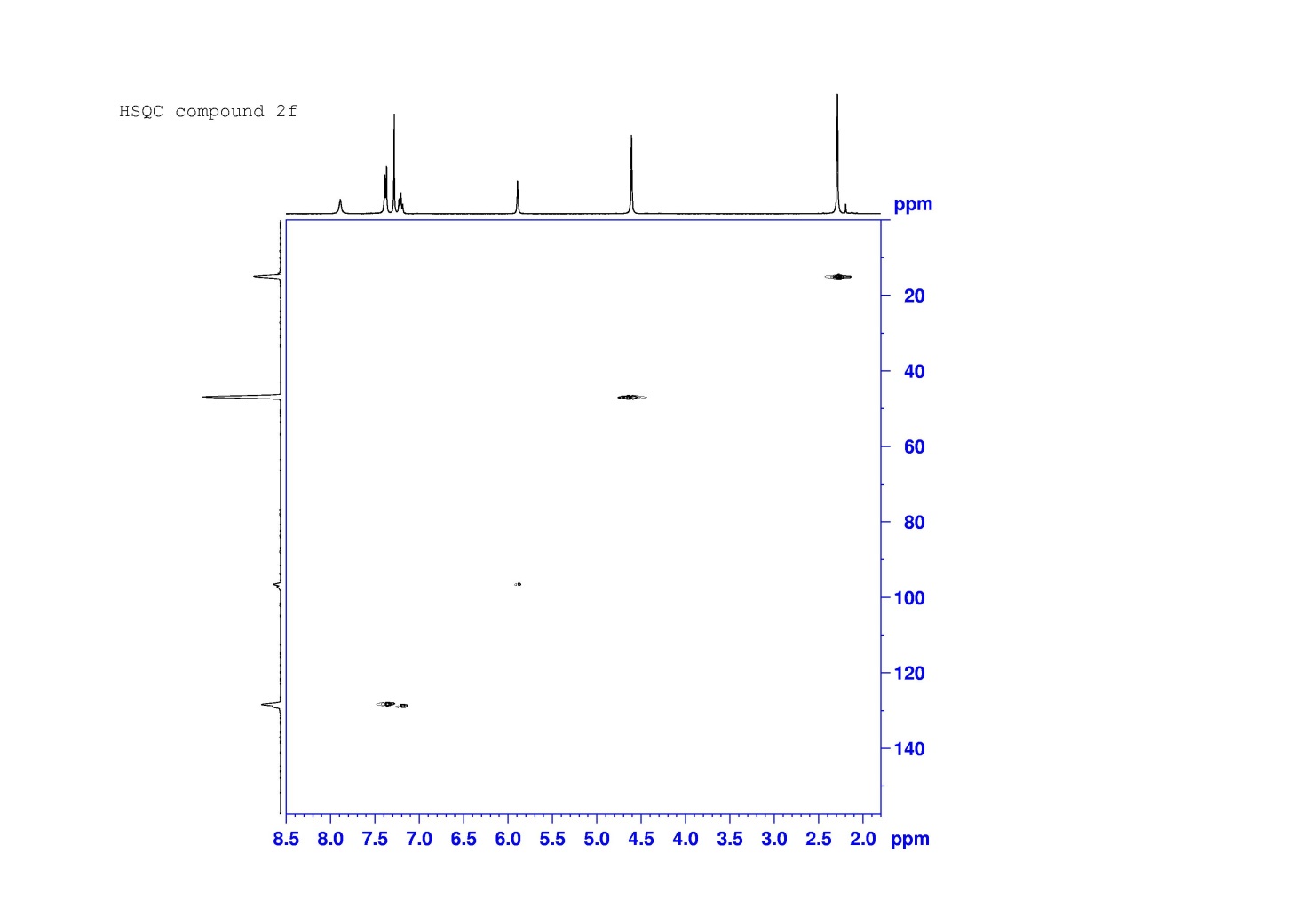

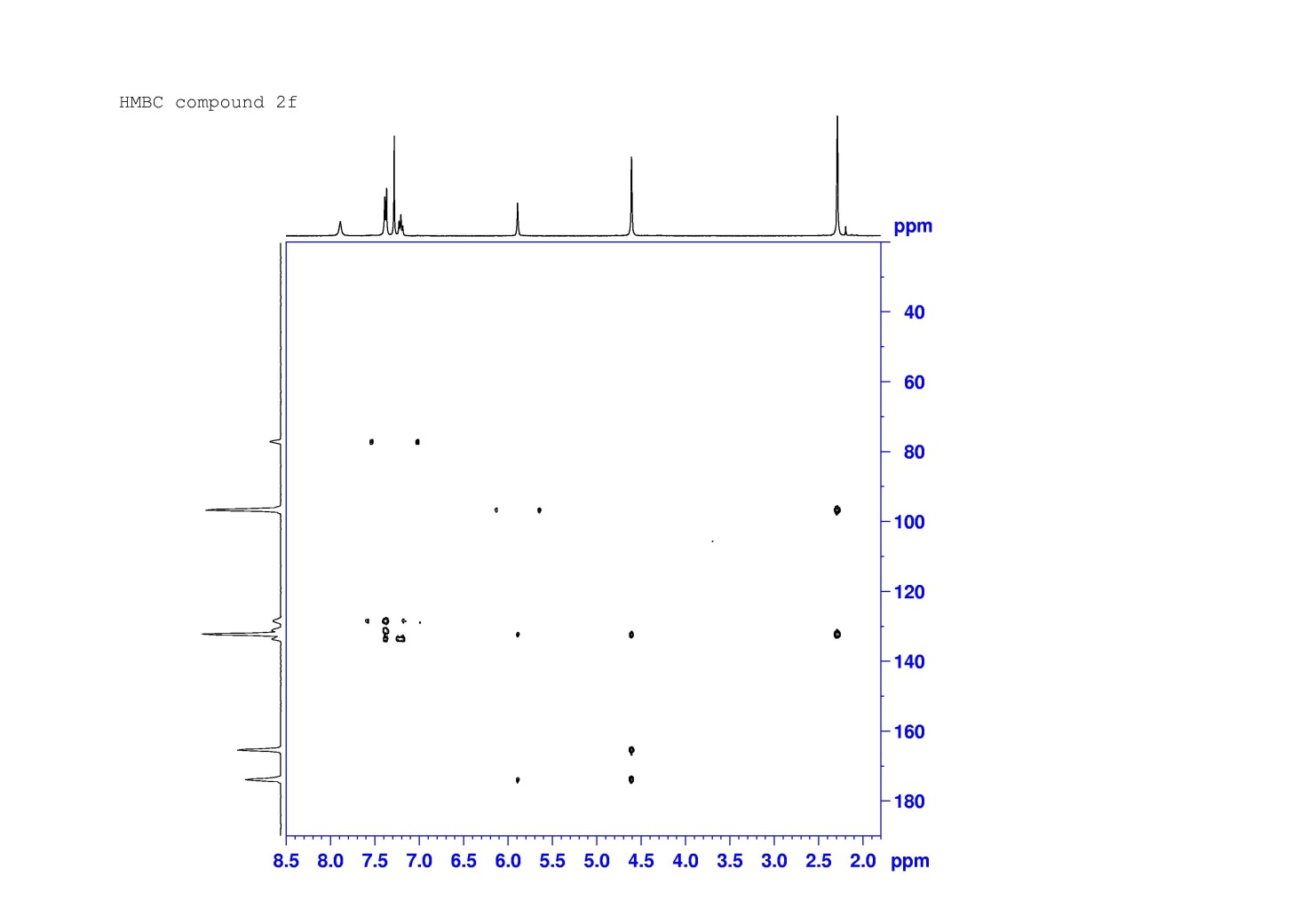


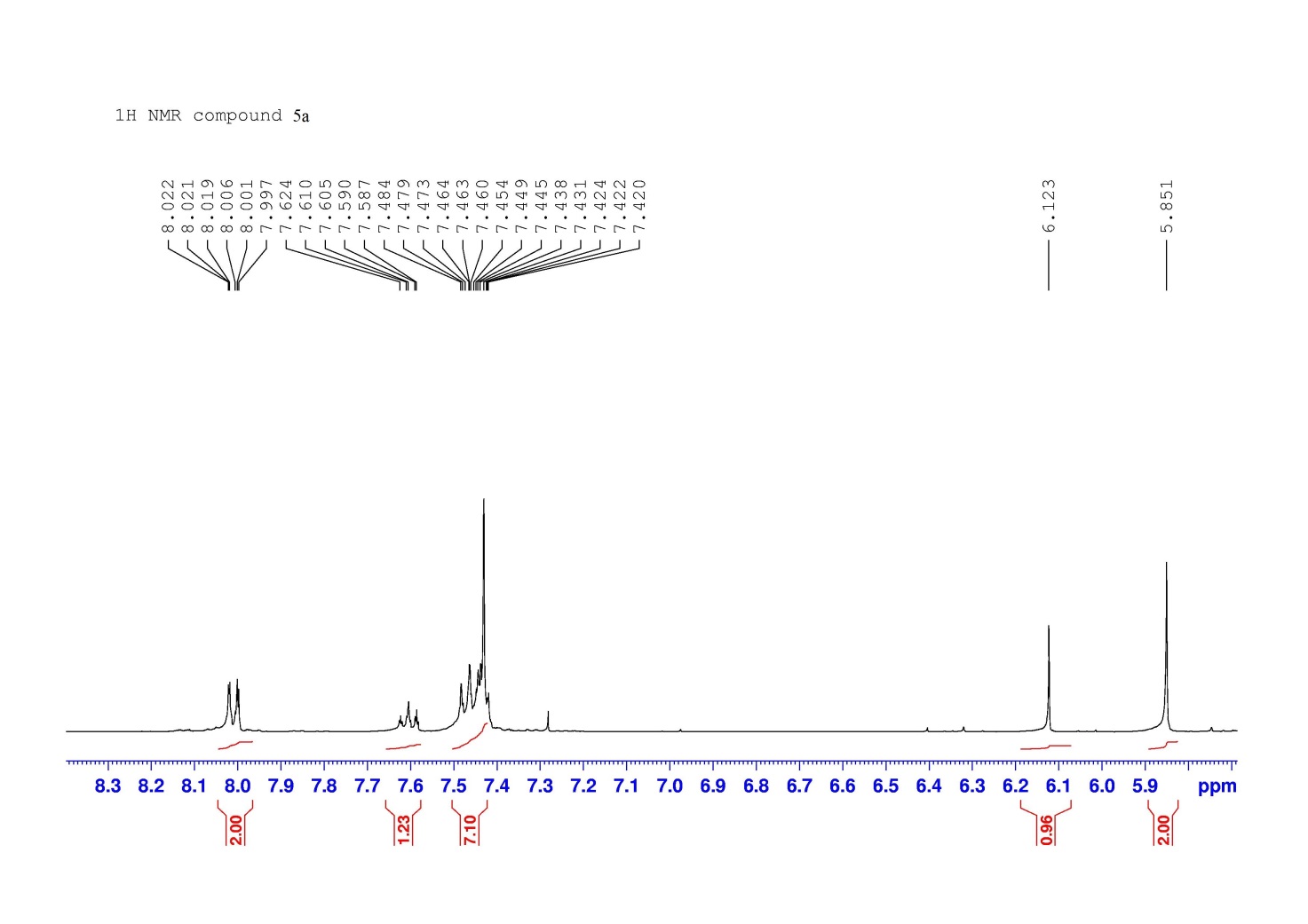


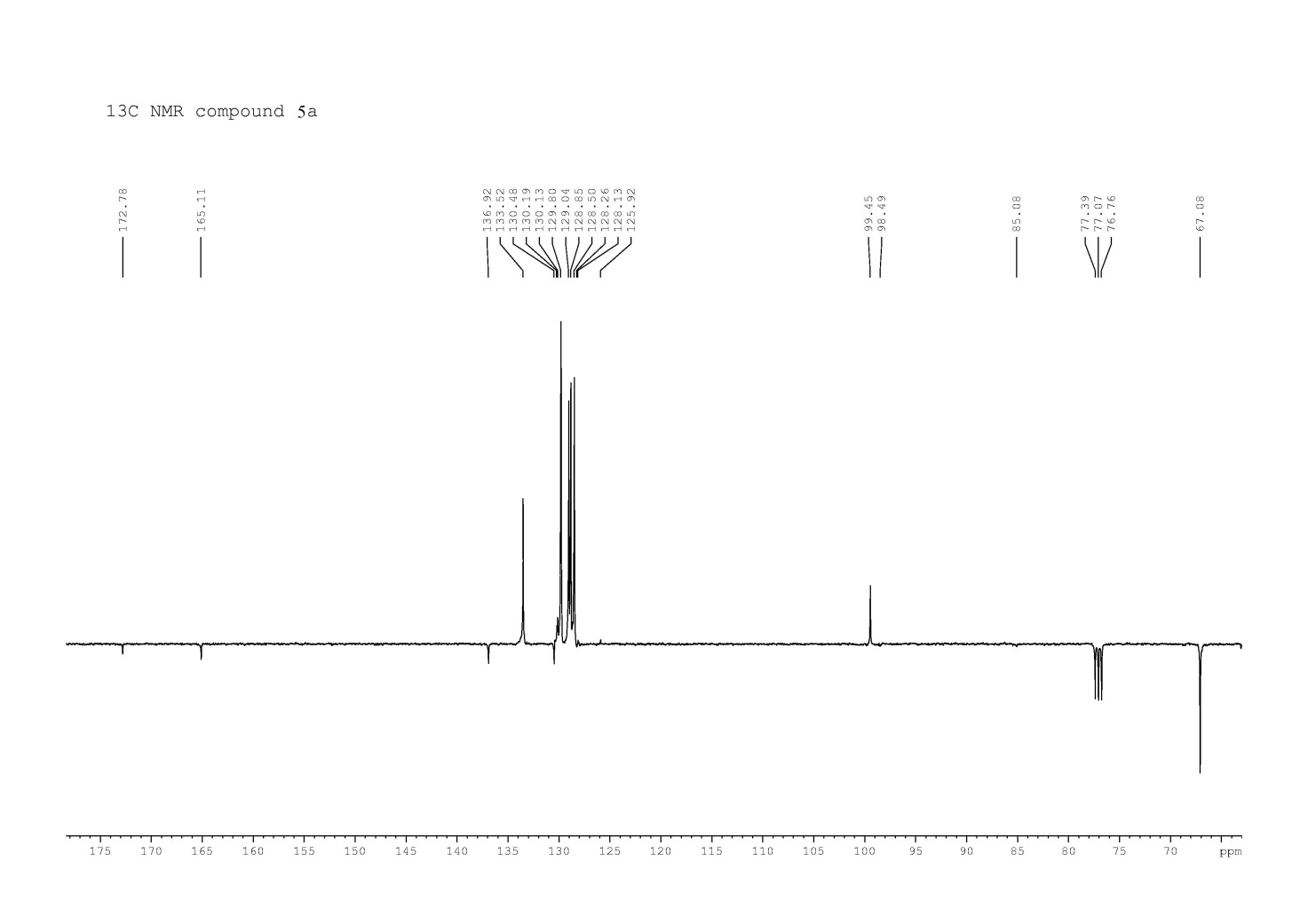


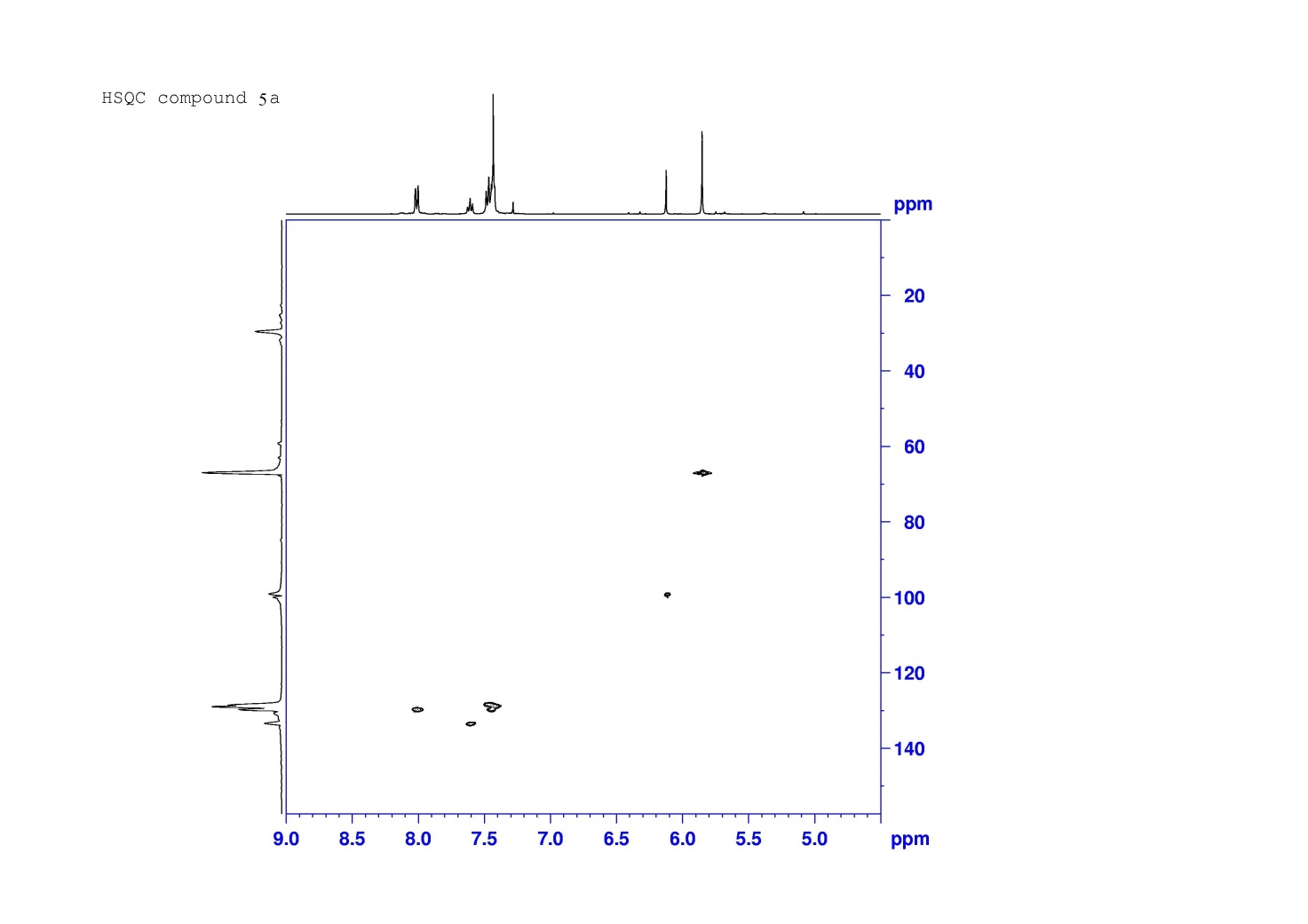


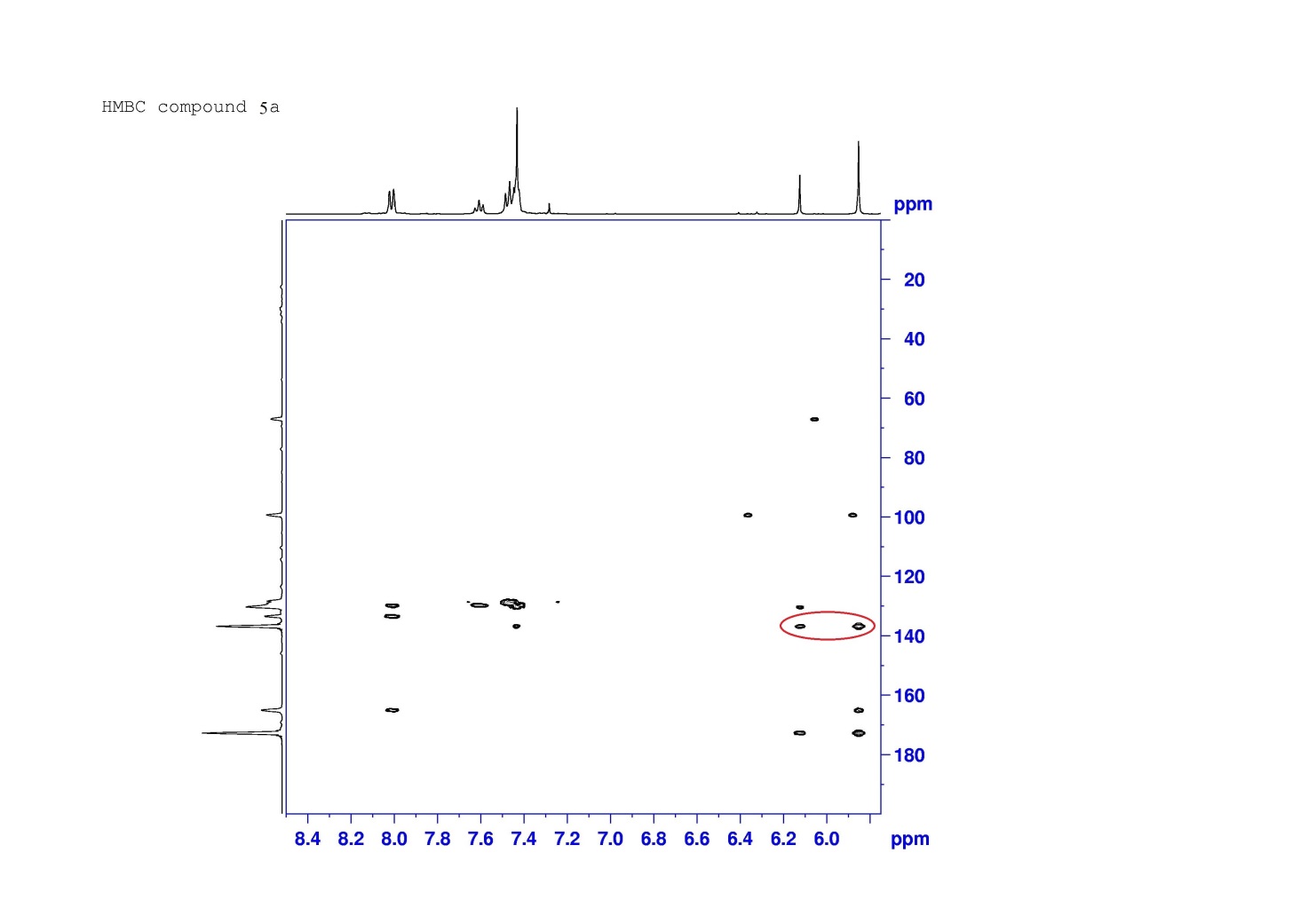


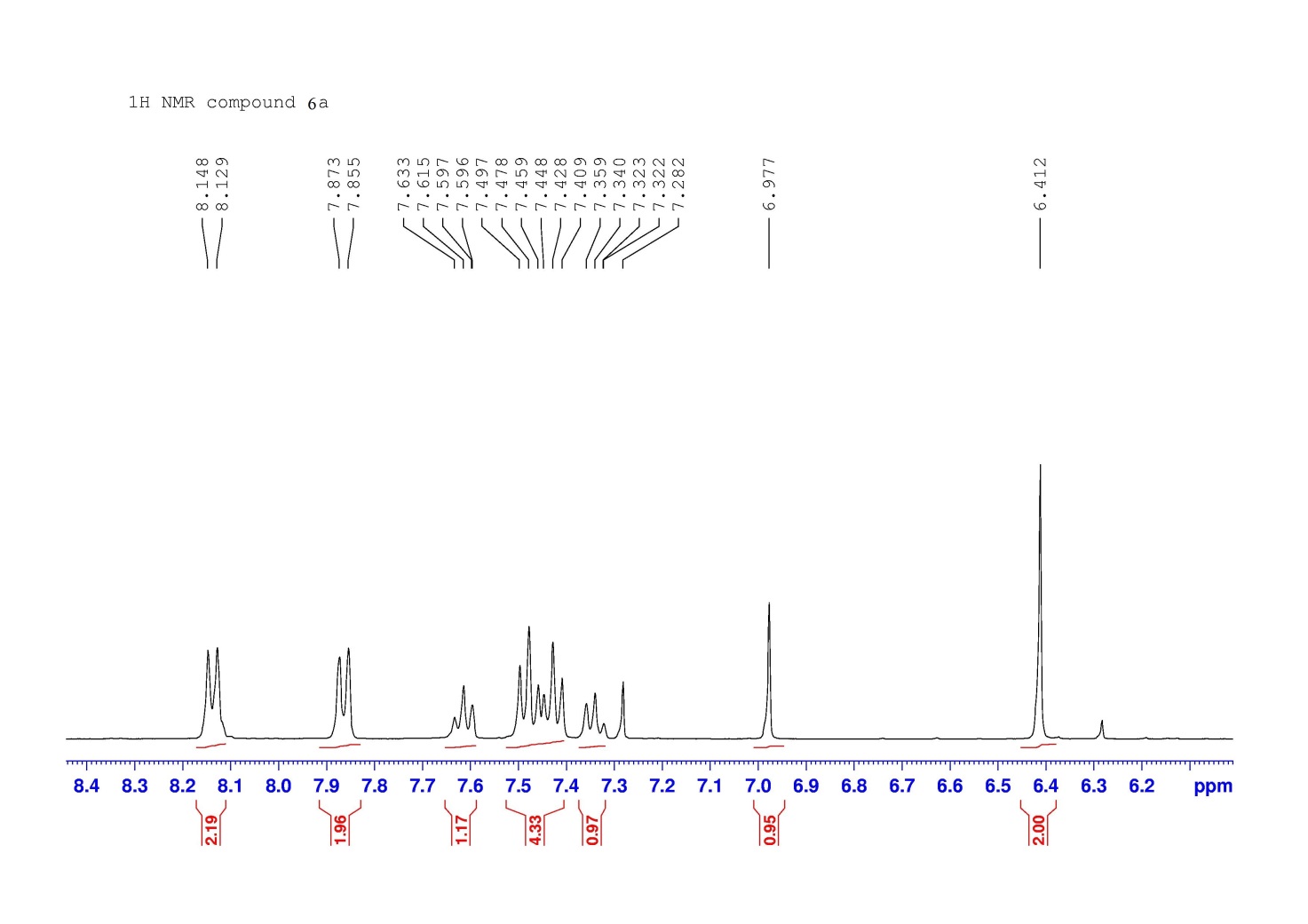


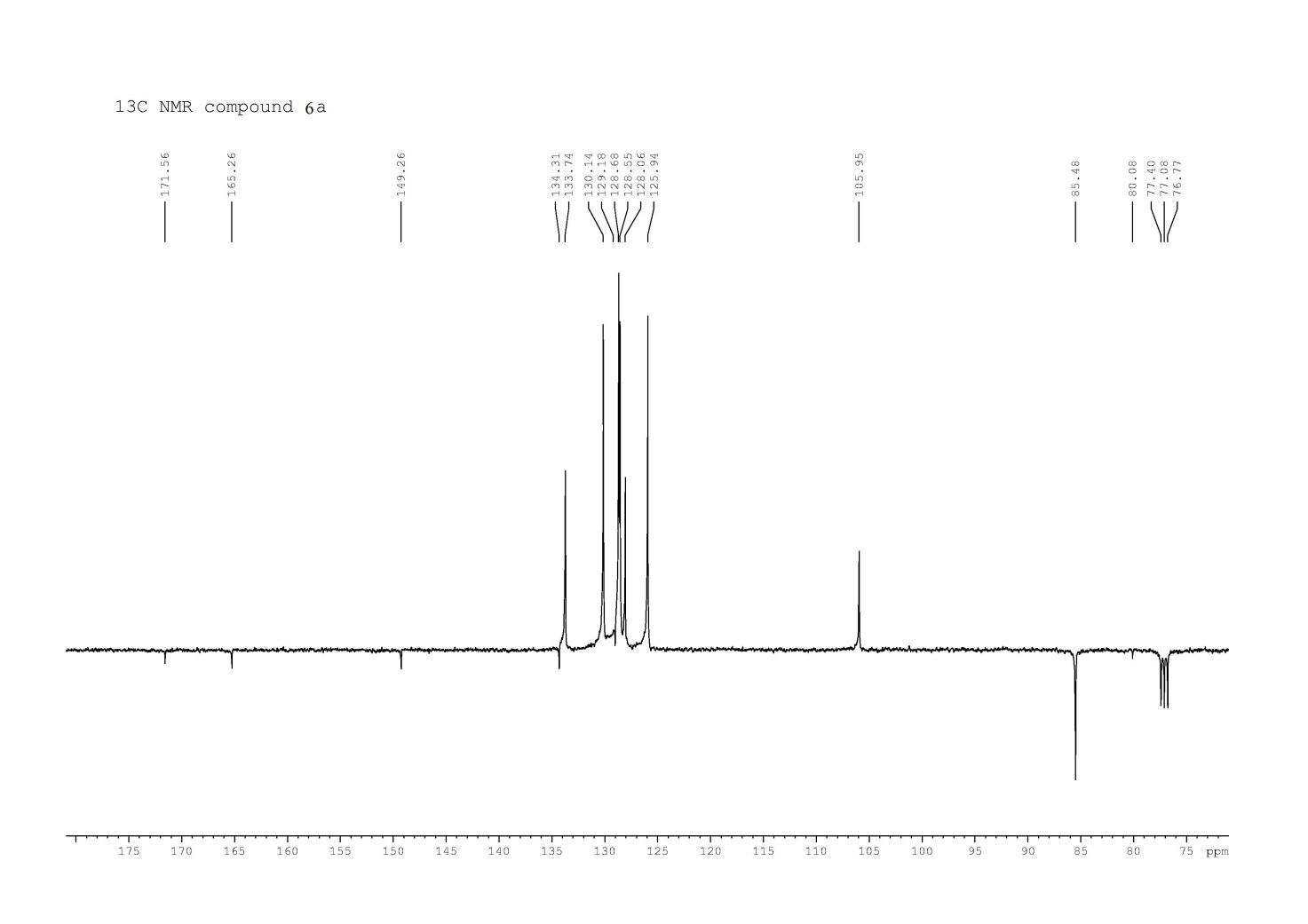


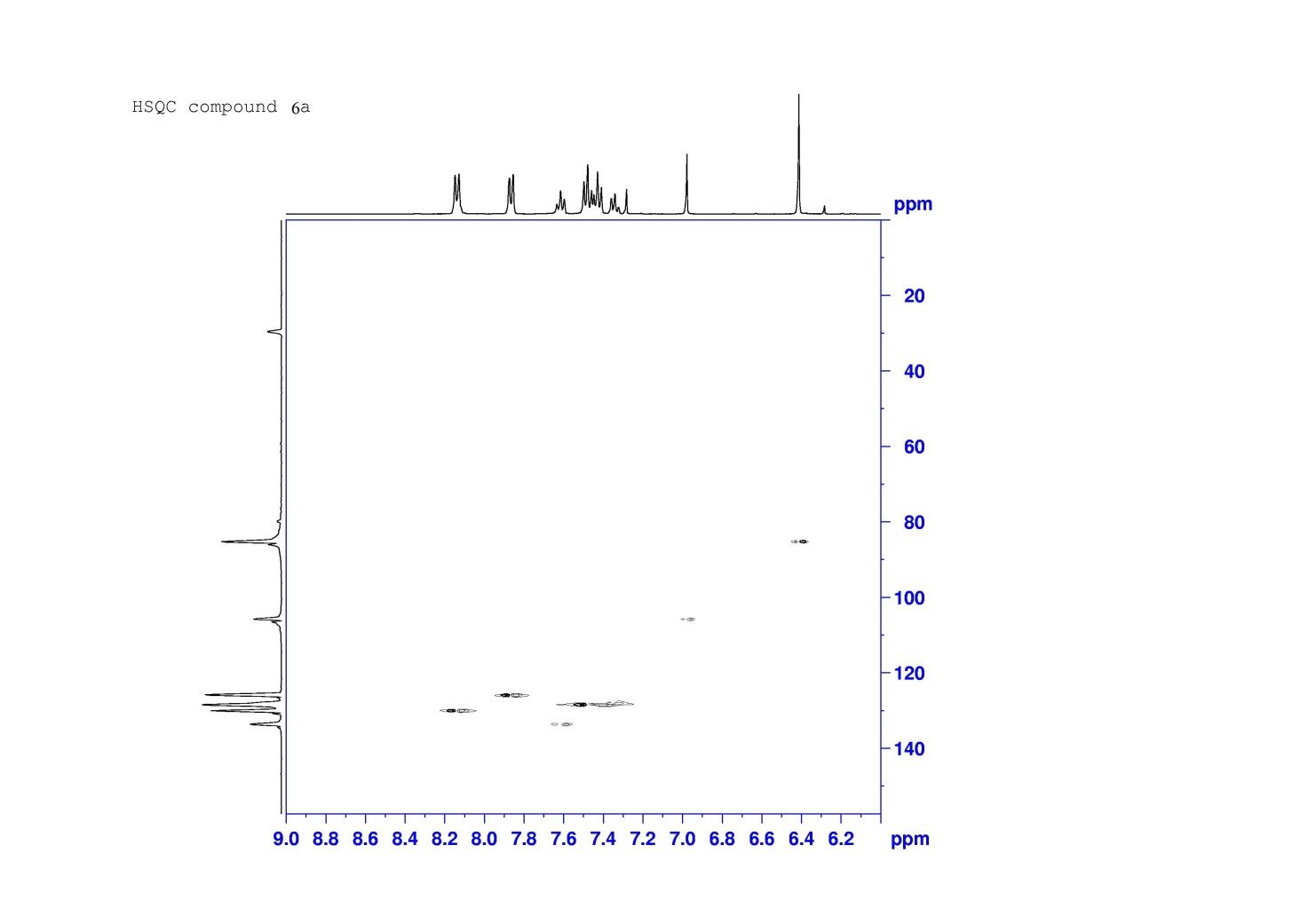


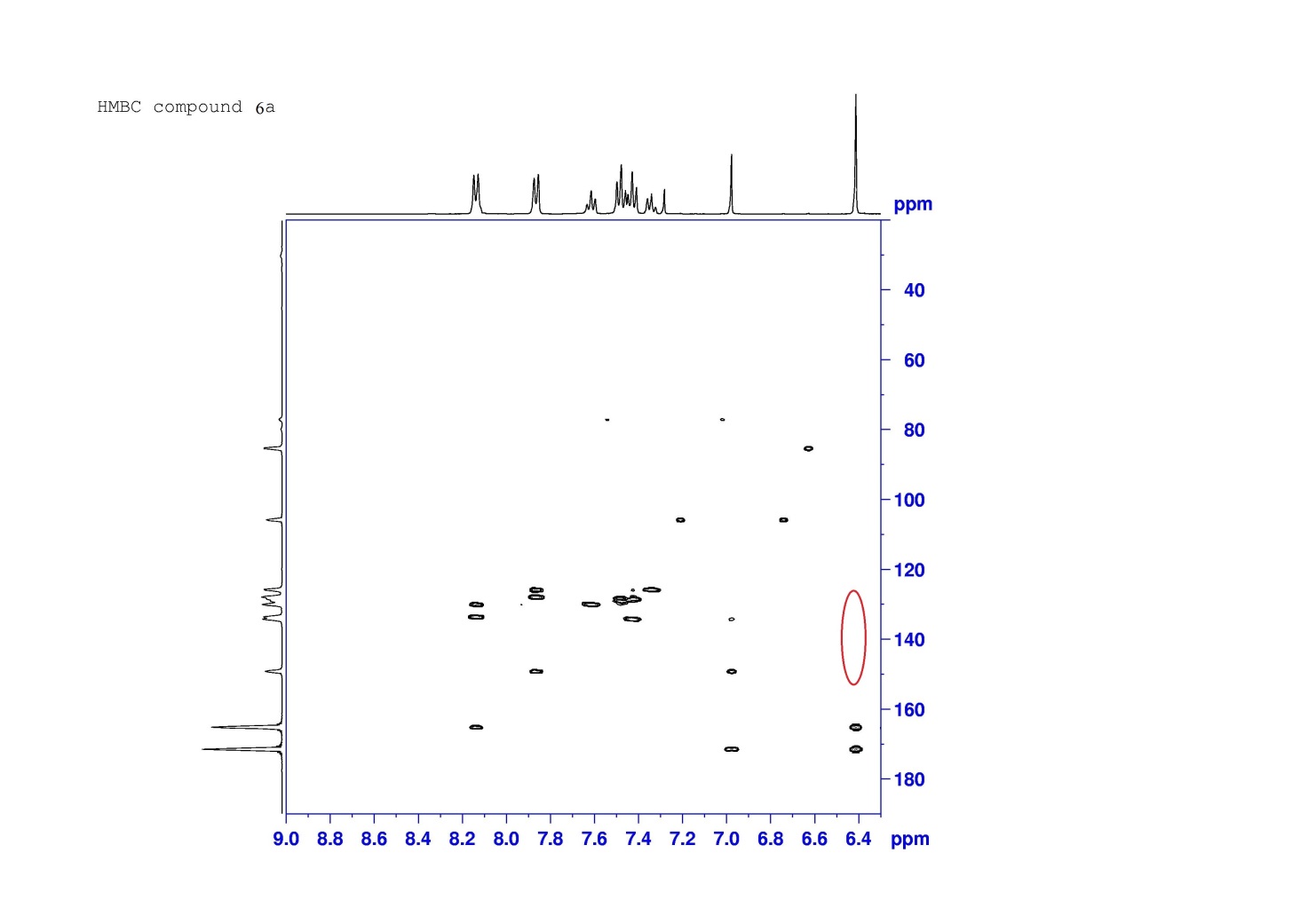


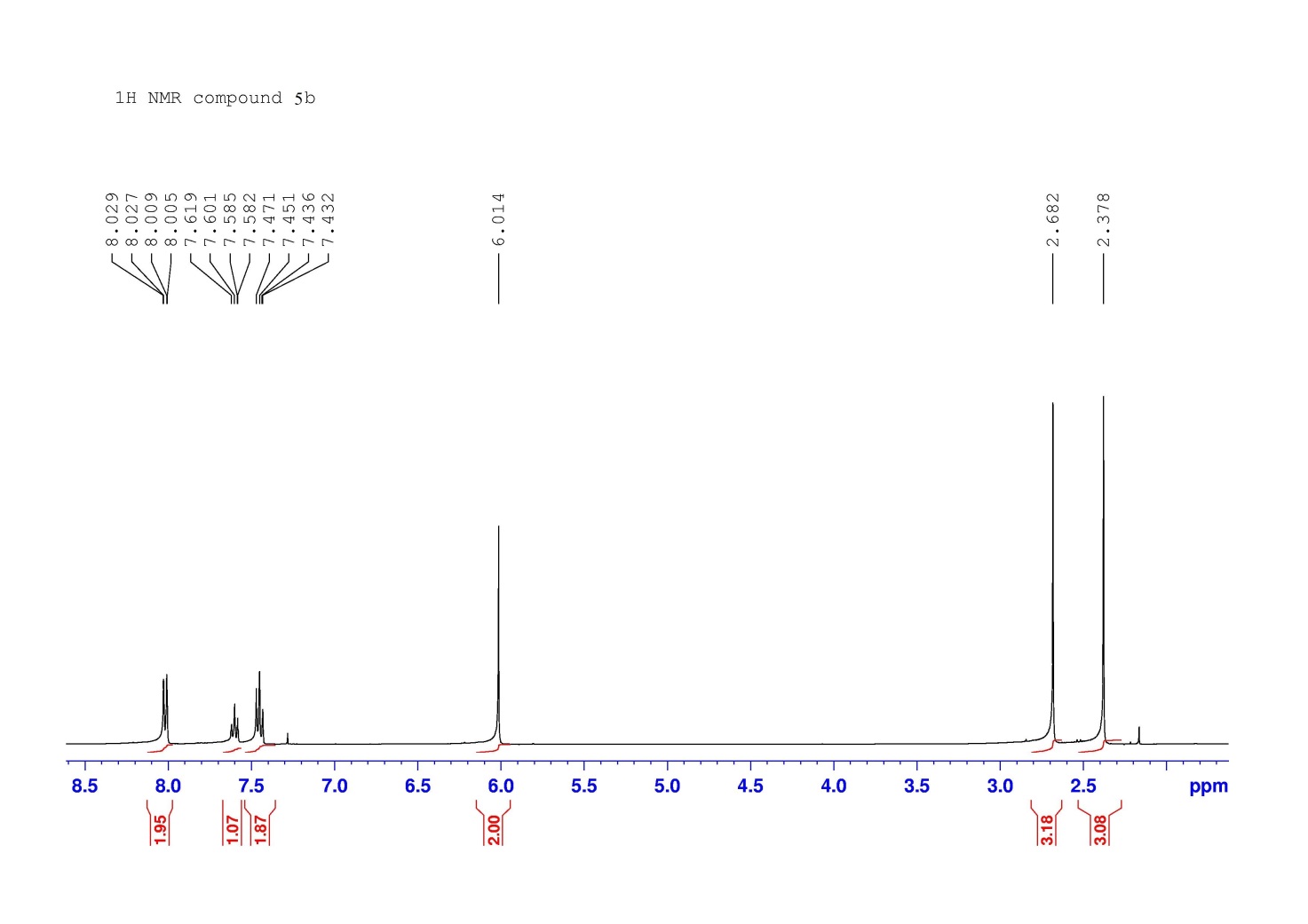


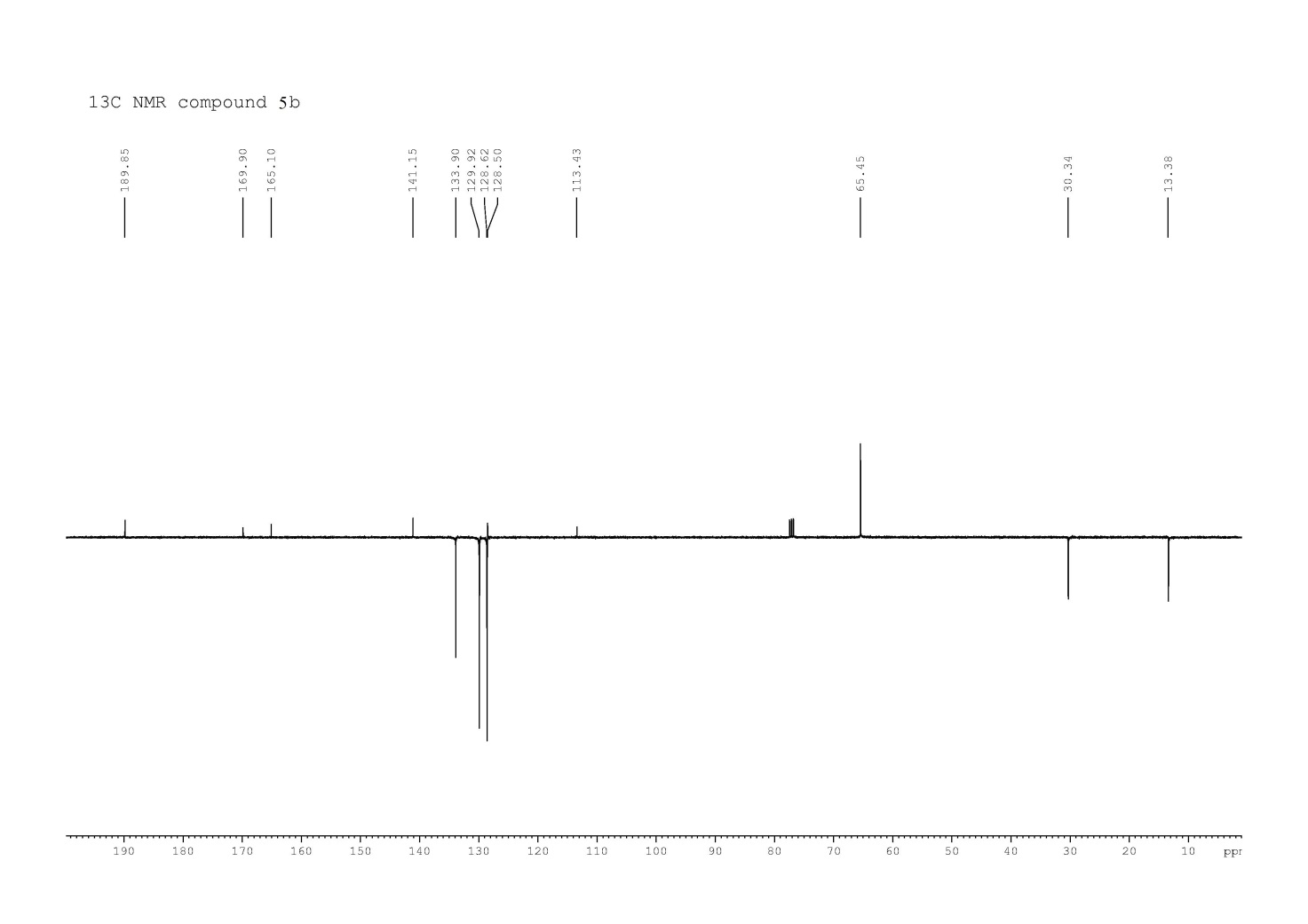


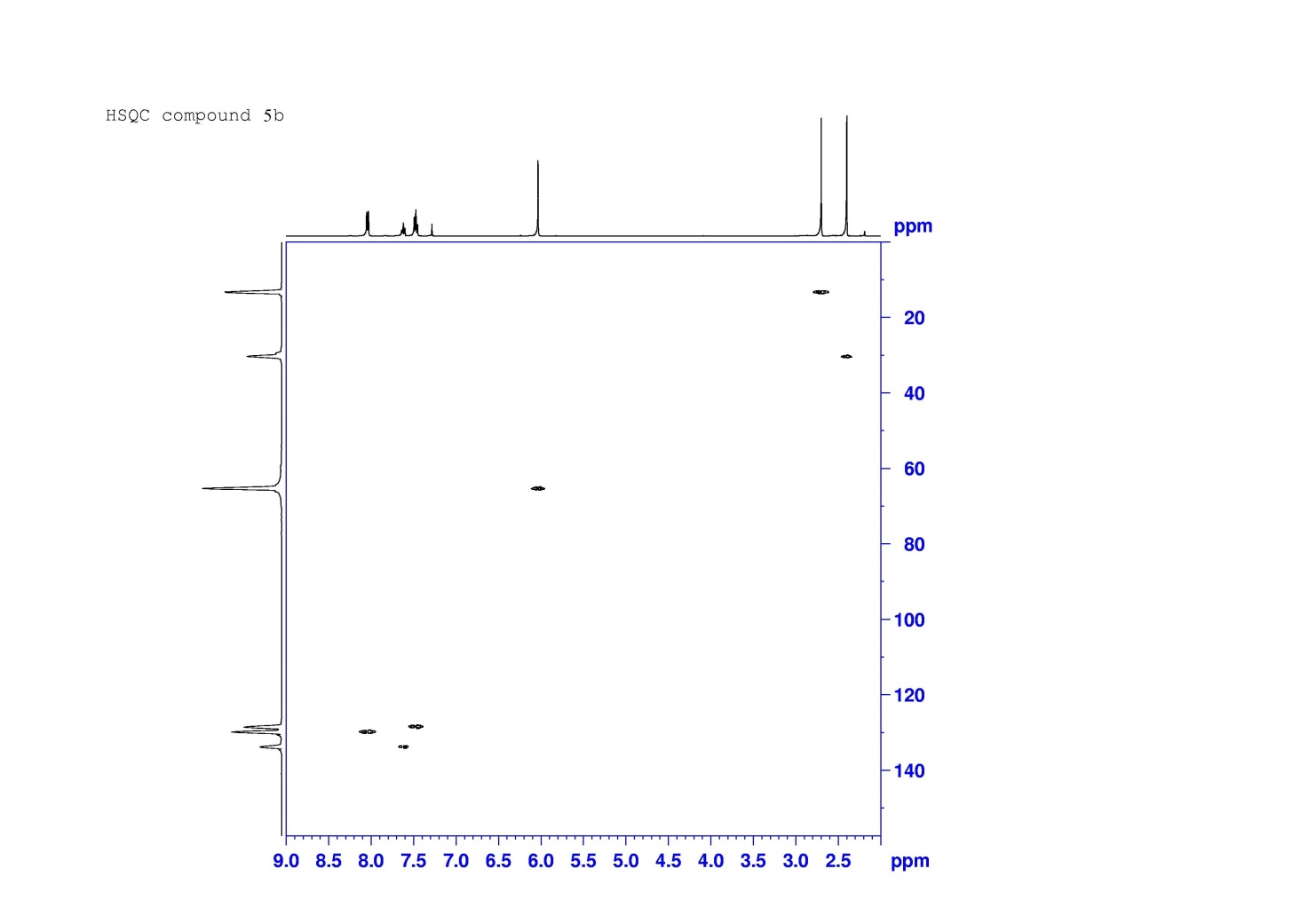


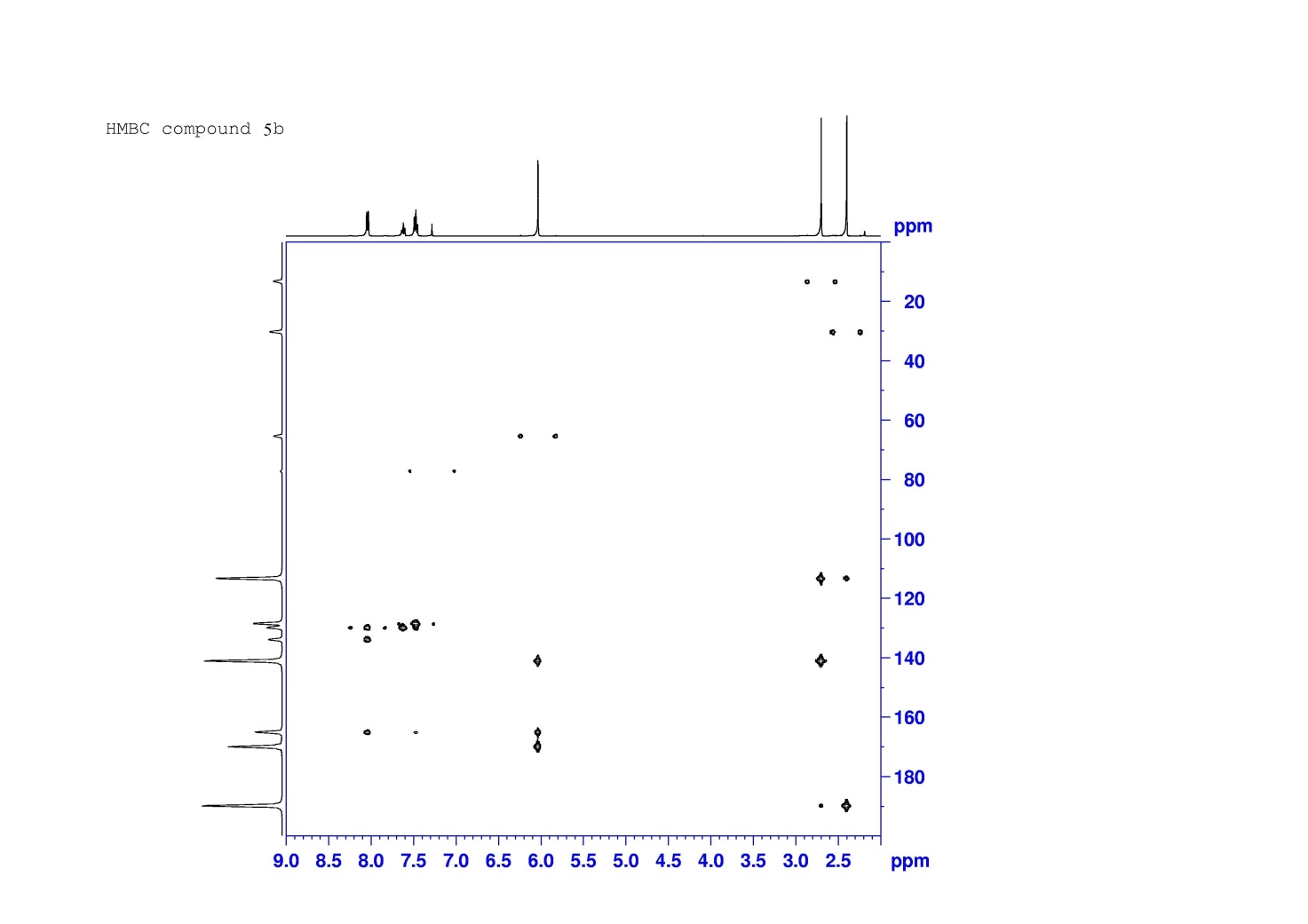


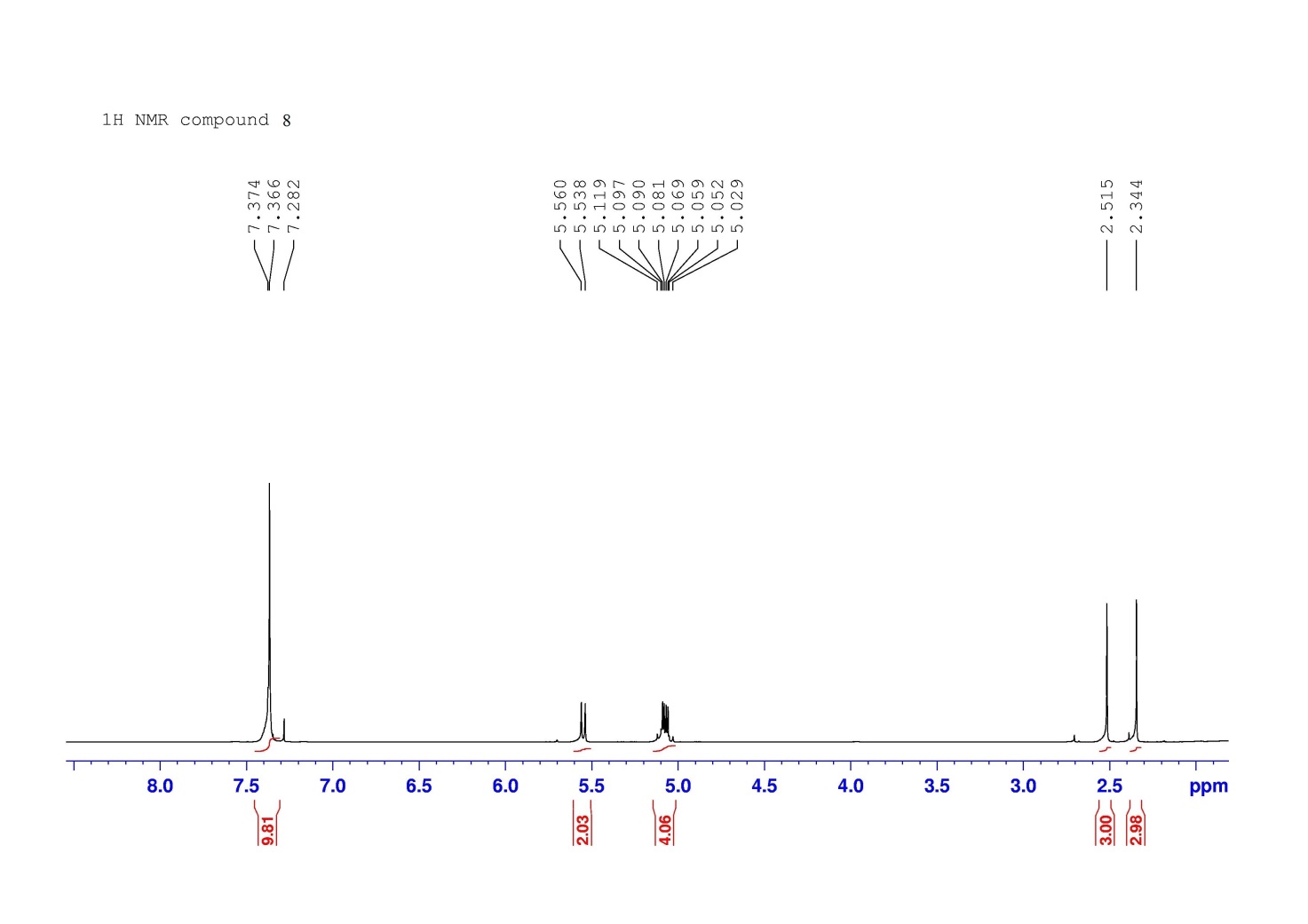


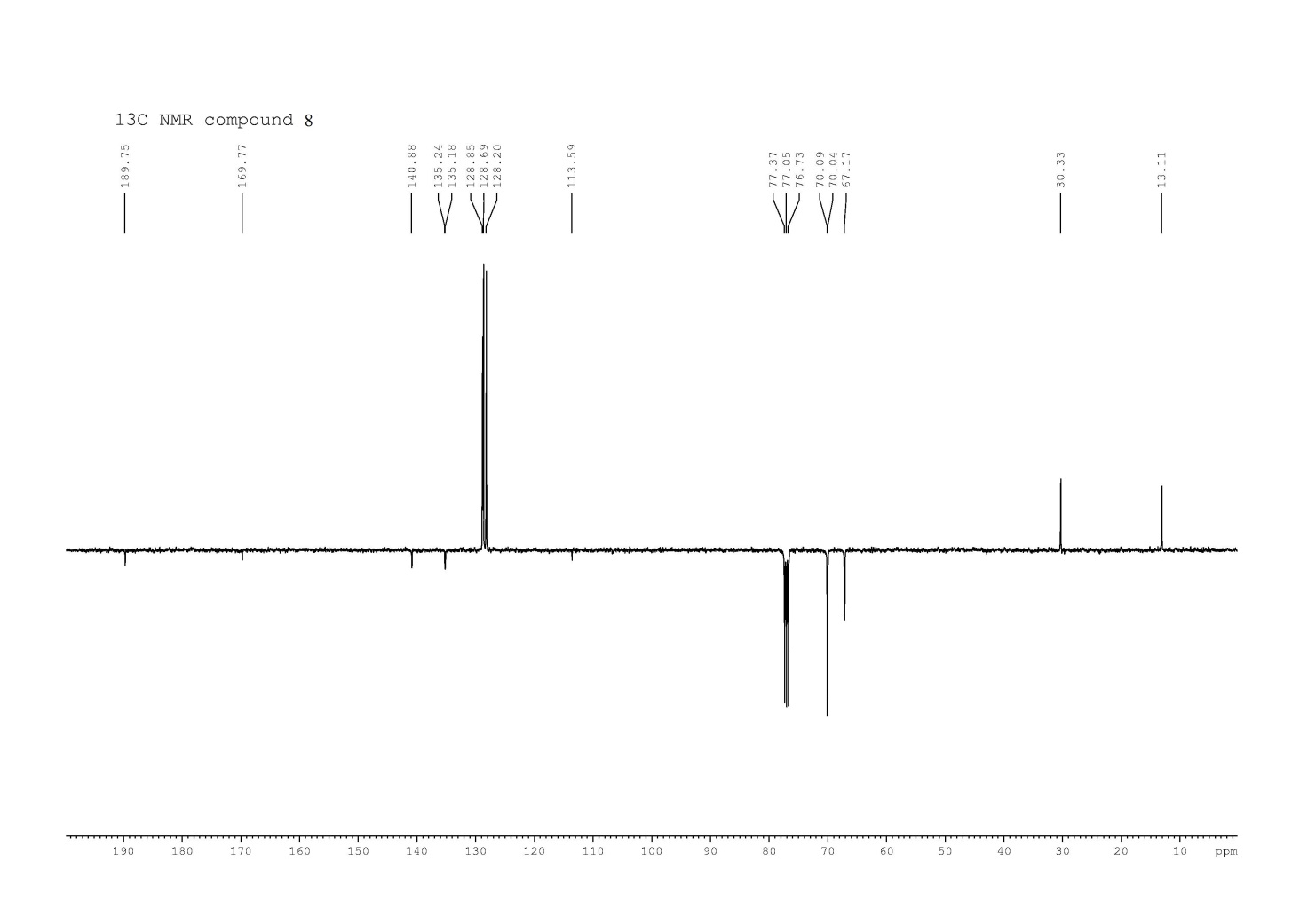


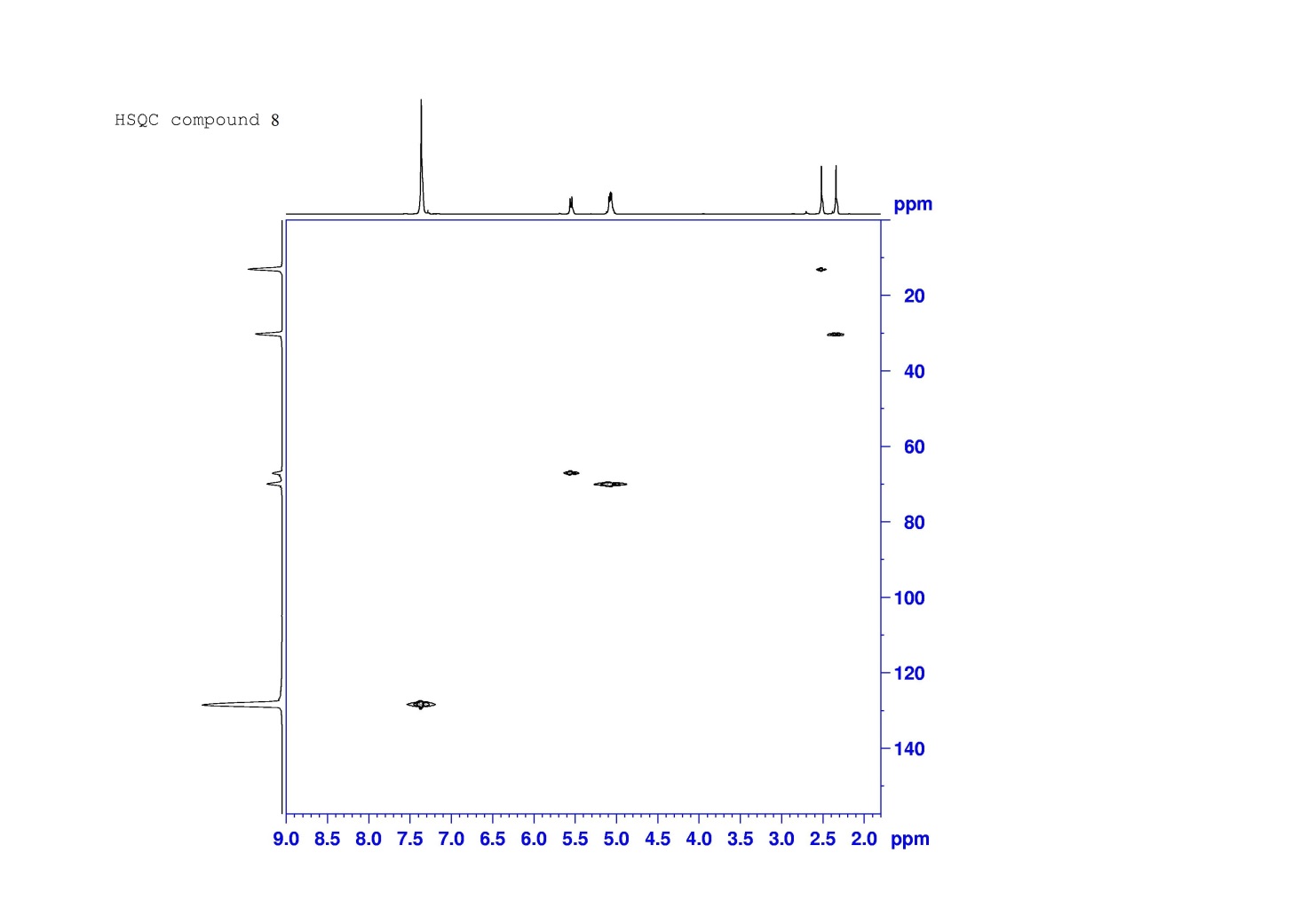


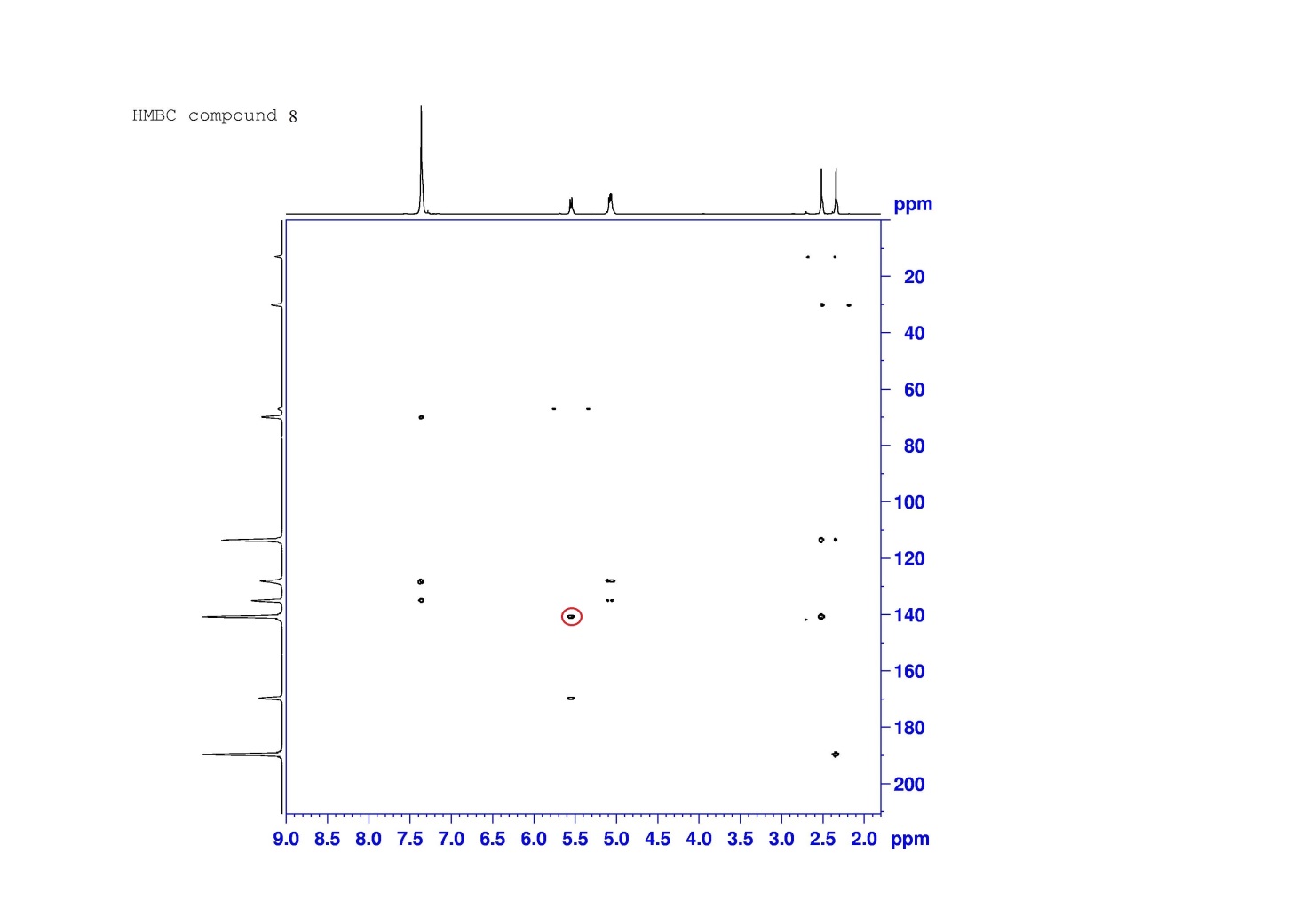


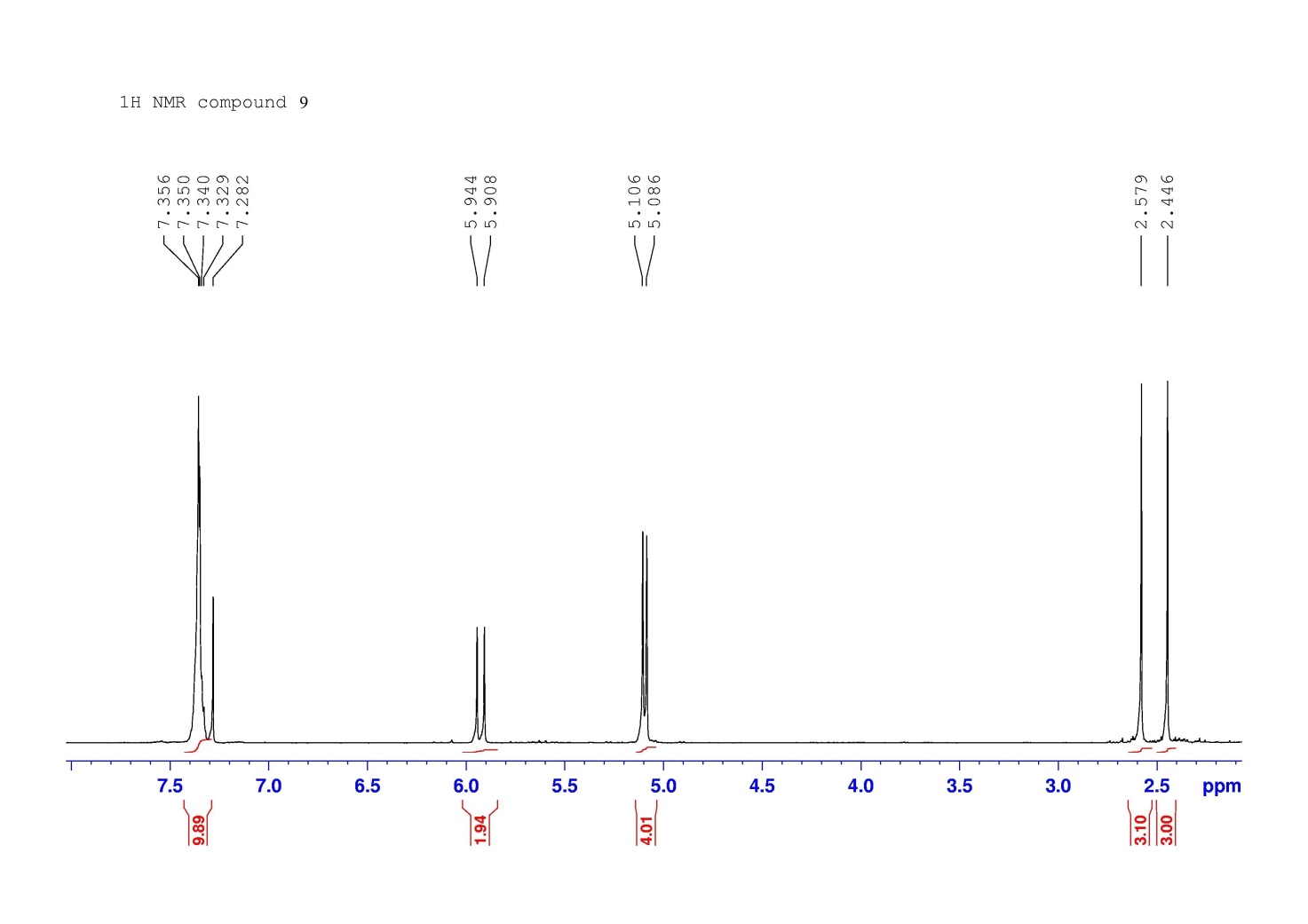


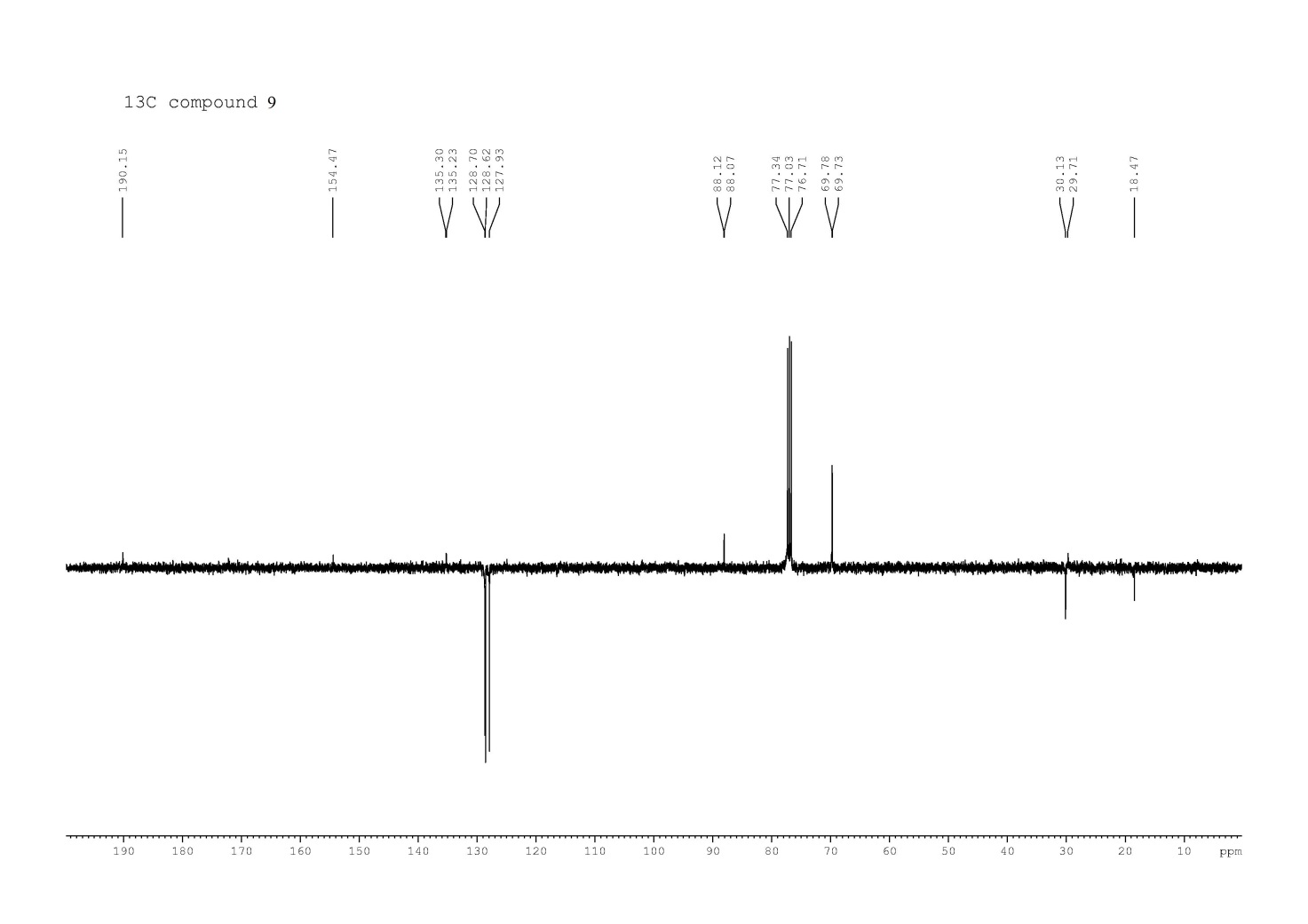


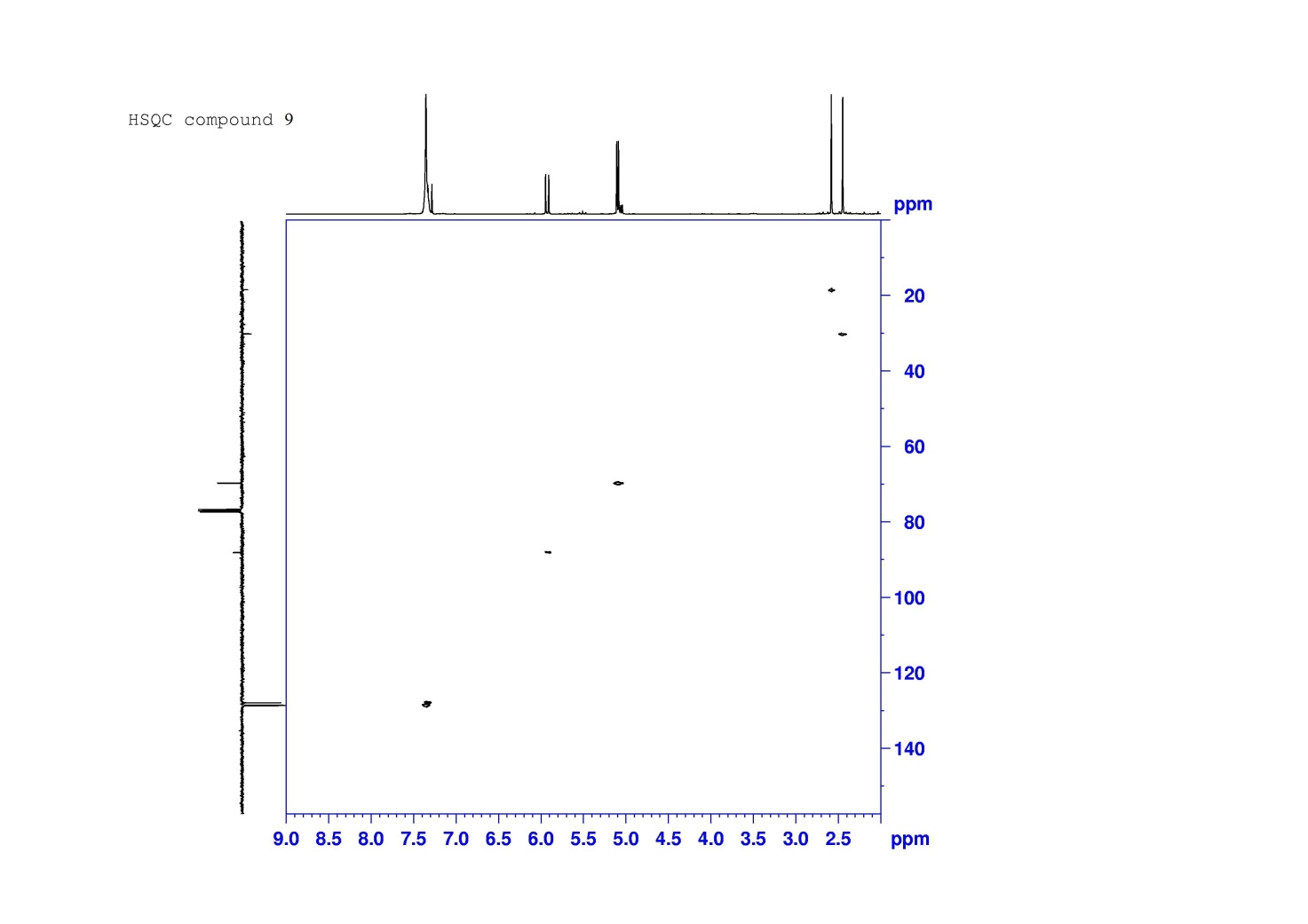


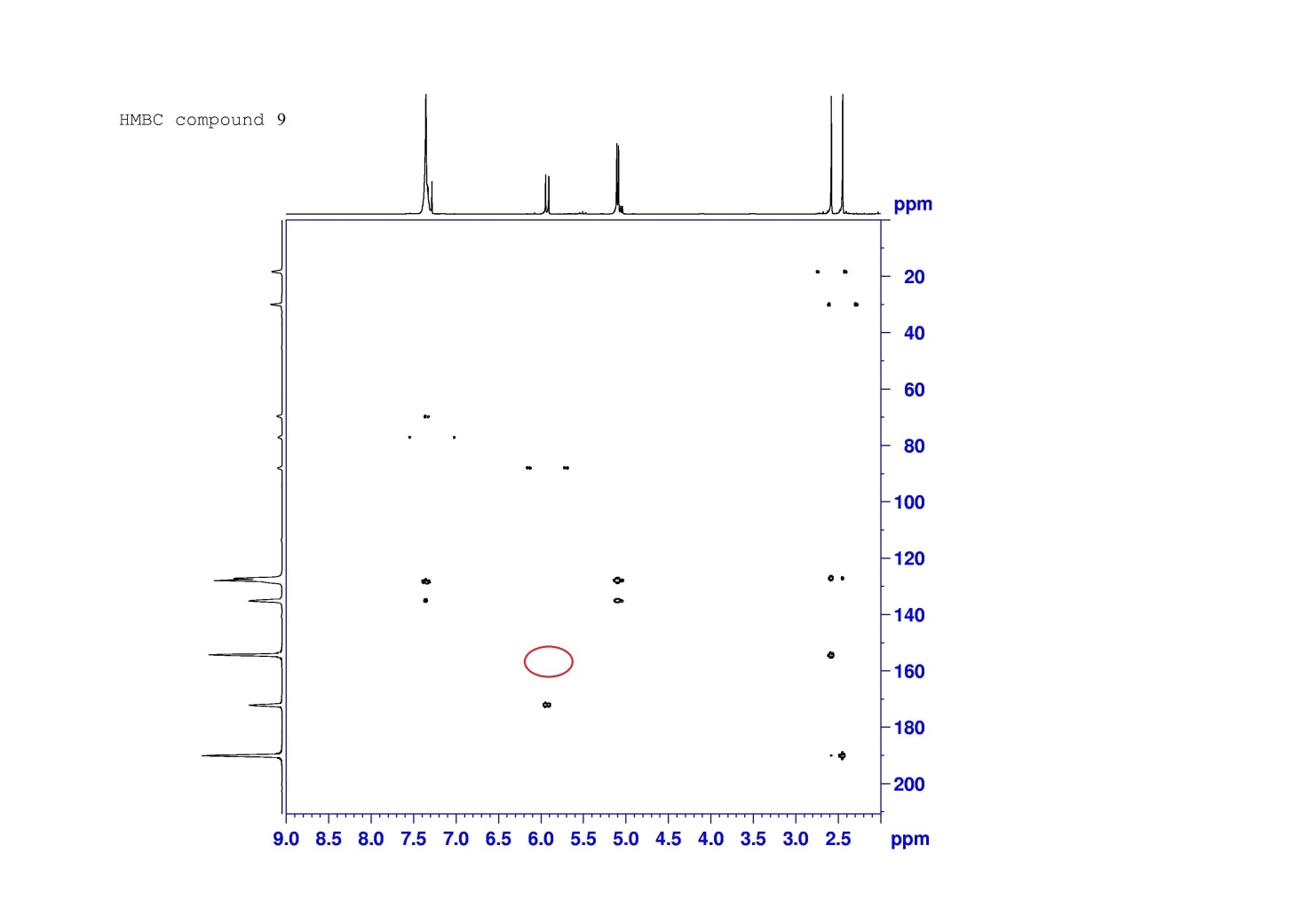


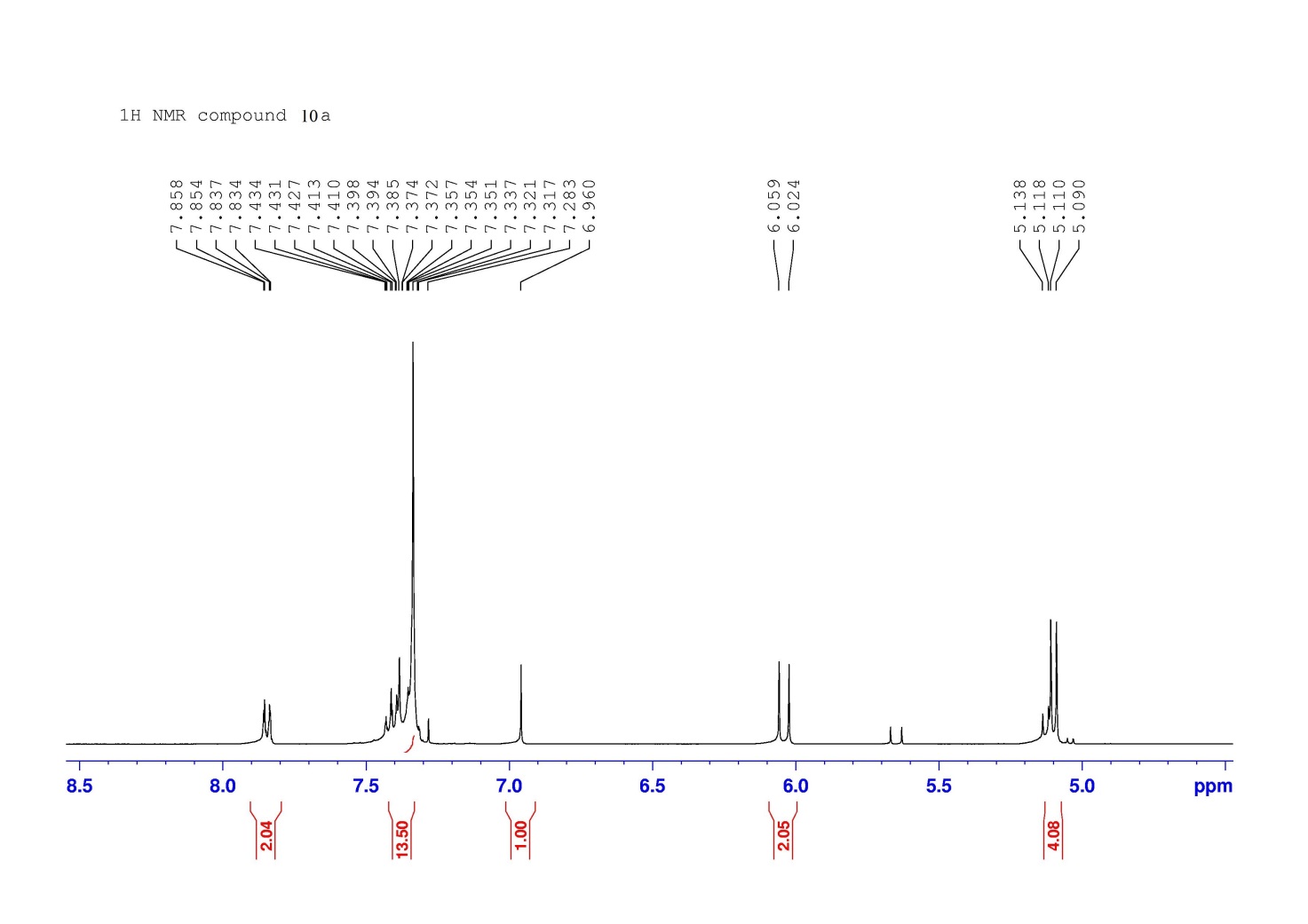


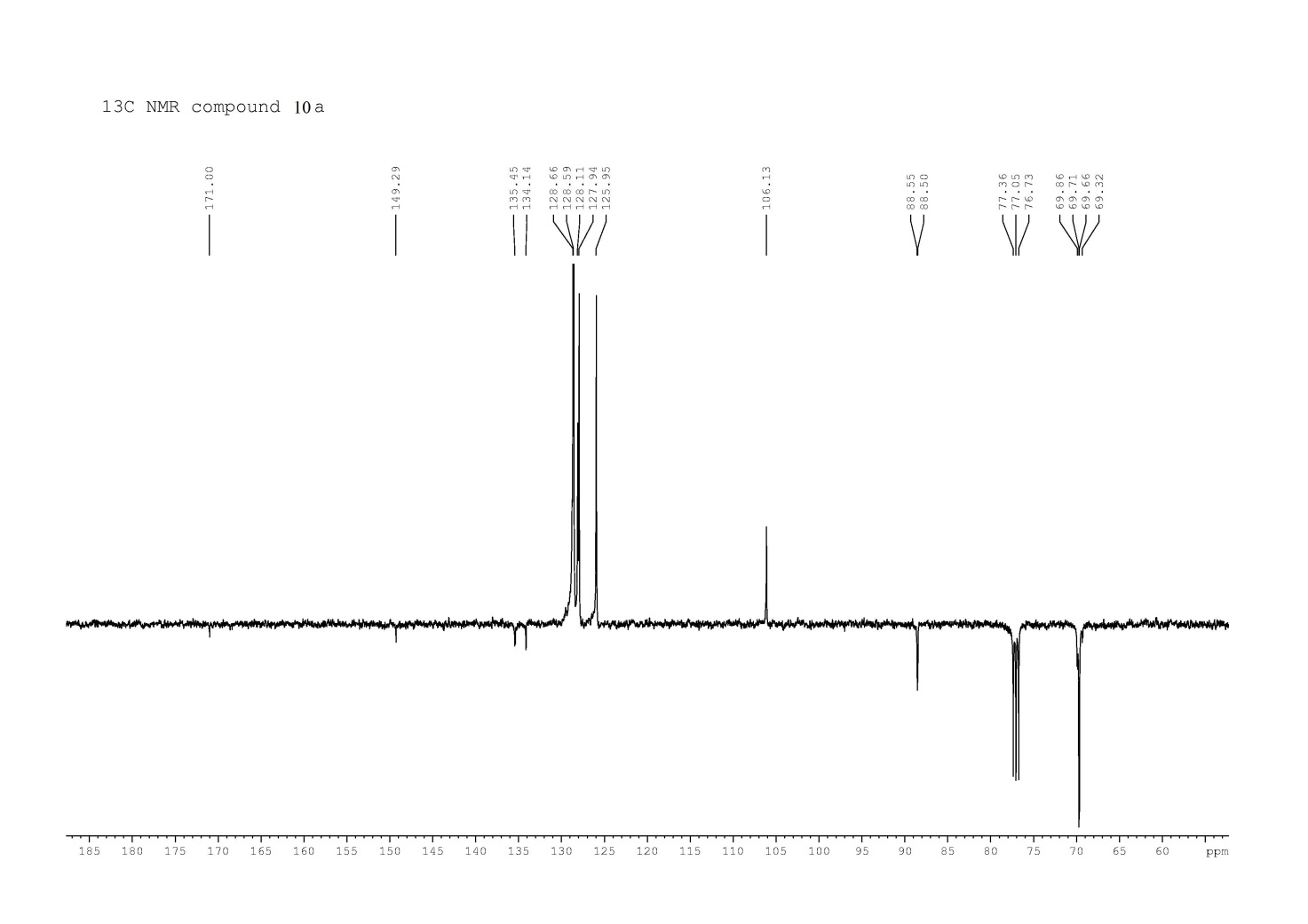


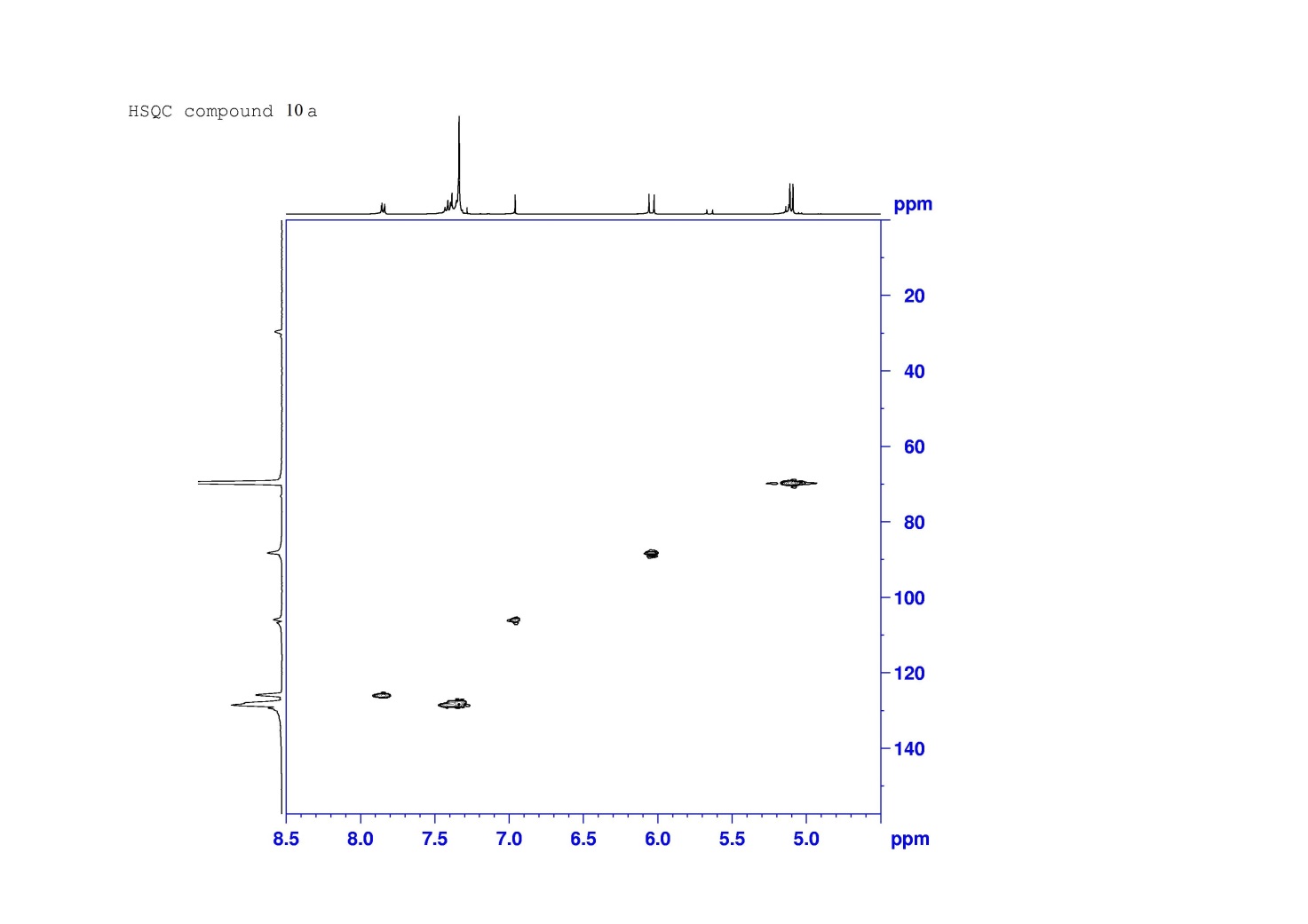


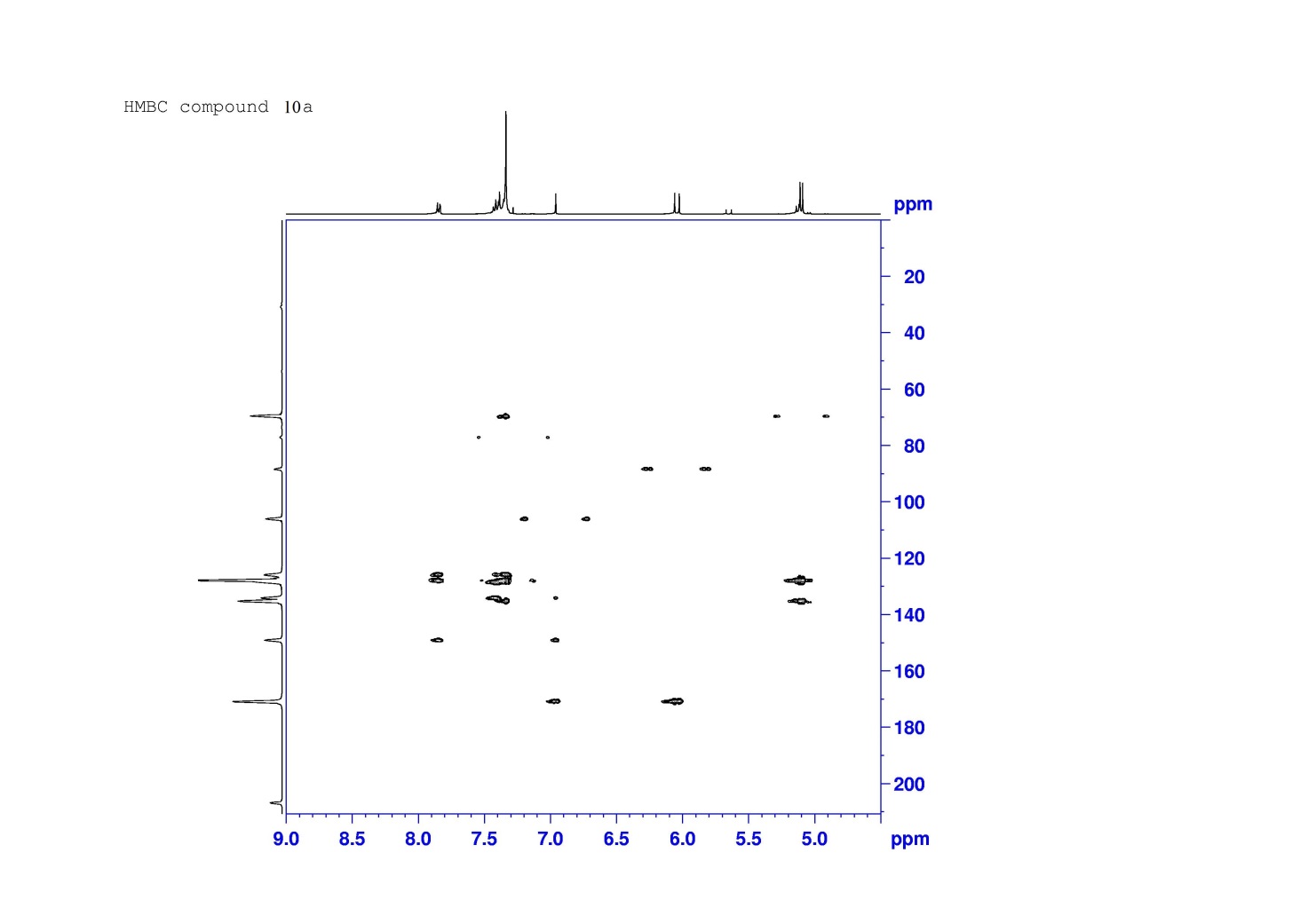


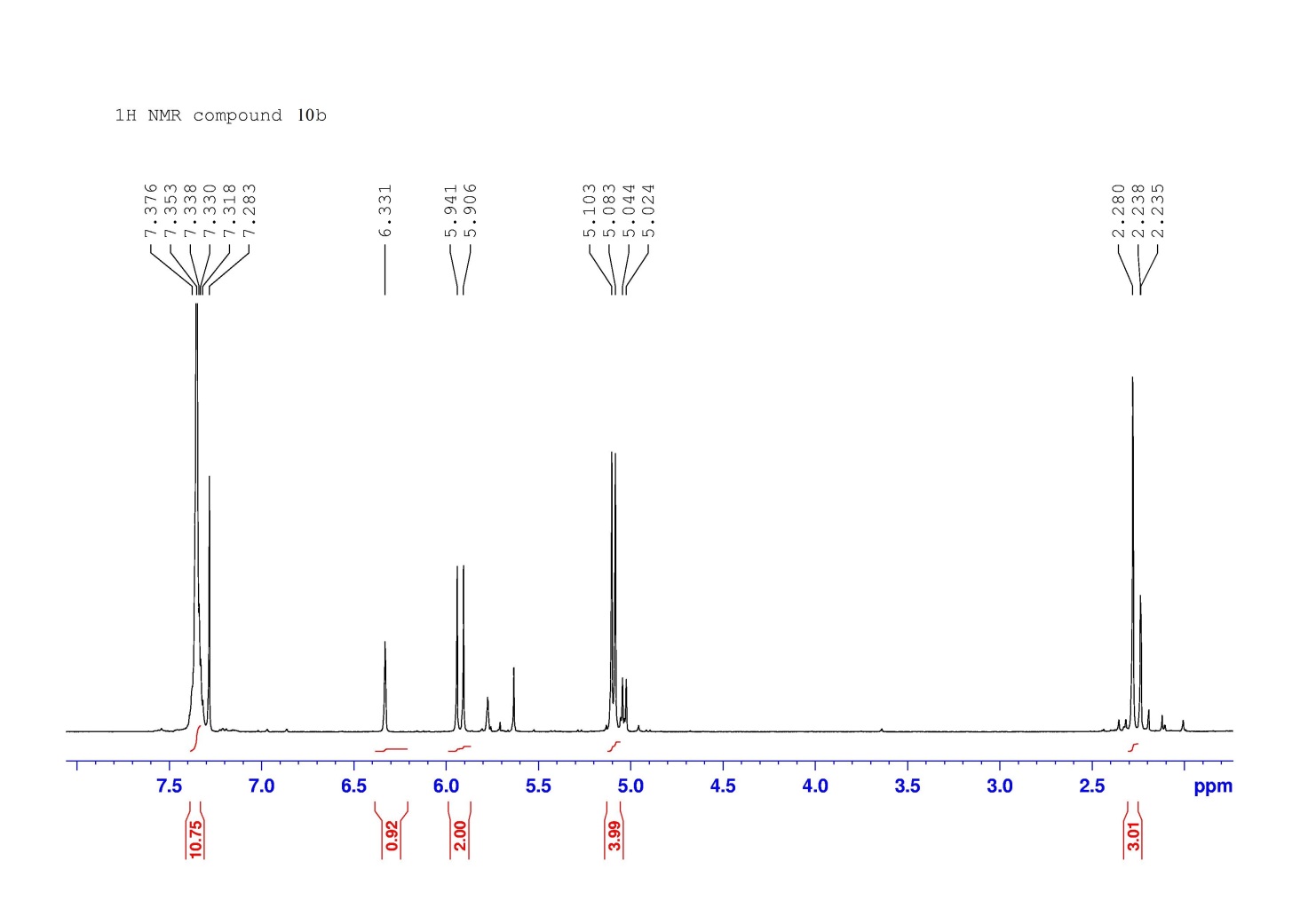


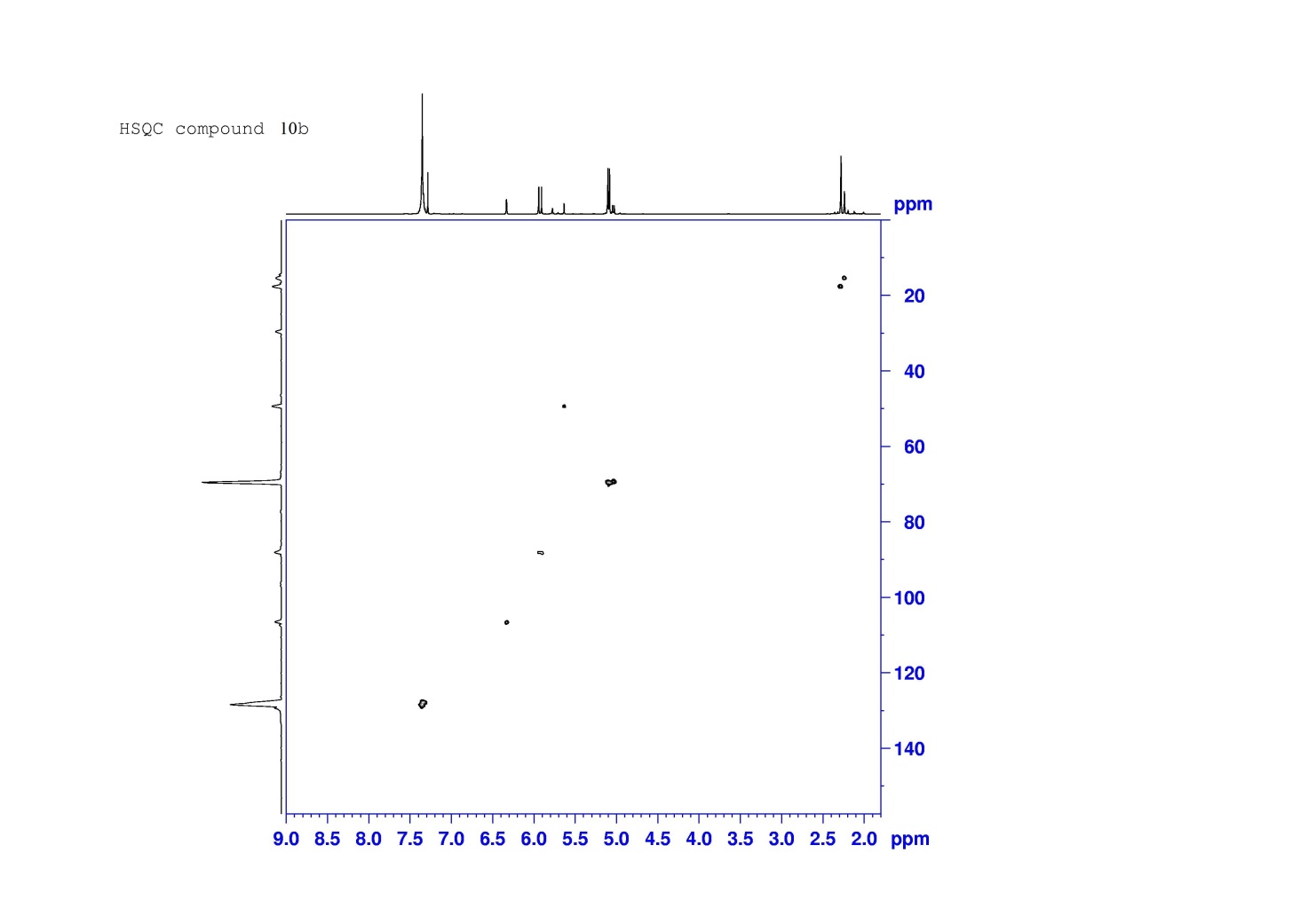


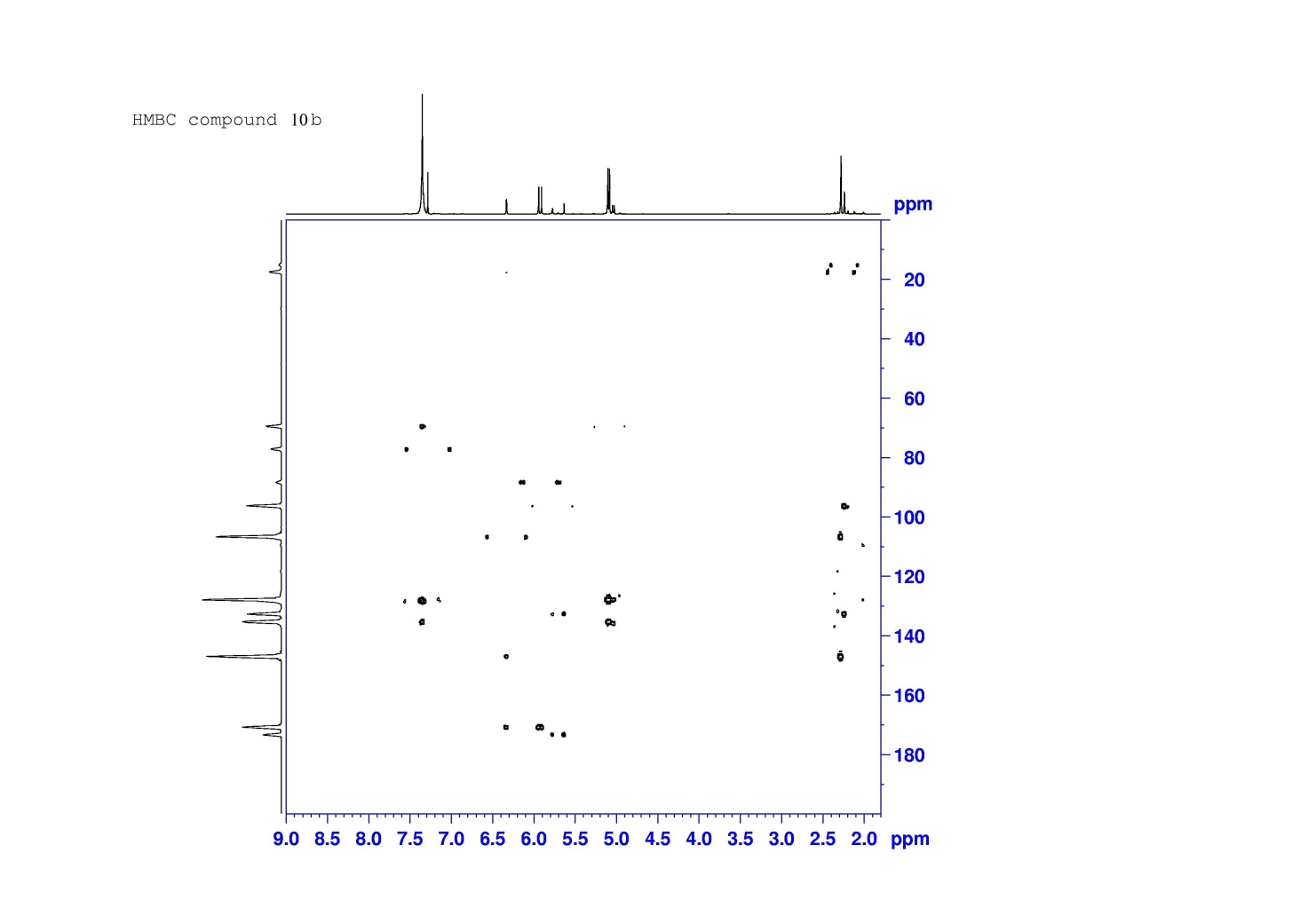


**Table S1.** Elemental analysis

| **Comp.** | **Formula (MW)** | **Anal. Calcd.** | | |  | **Anal. Found** | | |
| --- | --- | --- | --- | --- | --- | --- | --- | --- |
|  |  | **C** | **H** | **N** |  | **C** | **H** | **N** |
| **2a** | C_17_H_14_N_2_O_2_S (310.37) | 65.79 | 4.55 | 9.03 |  | 66.05 | 4.57 | 9.06 |
| **2b** | C_17_H_12_Cl_2_N_2_O_2_S (379.26) | 53.84 | 3.19 | 7.39 |  | 54.05 | 3.20 | 7.41 |
| **2c** | C_14_H_14_N_2_O_3_S (290.34) | 57.92 | 4.86 | 9.65 |  | 58.15 | 4.88 | 9.68 |
| **2d** | C_14_H_12_Cl_2_N_2_O_3_S (359.23) | 46.81 | 3.37 | 7.80 |  | 46.99 | 3.38 | 7.83 |
| **2e** | C_12_H_12_N_2_O_2_S (248.30) | 58.05 | 4.87 | 11.28 |  | 58.28 | 4.89 | 11.32 |
| **2f** | C_12_H_10_Cl_2_N_2_O_2_S (317.19) | 45.44 | 3.18 | 8.83 |  | 45.62 | 3.19 | 8.86 |
| **3a** | C_16_H_13_NOS (267.35) | 71.88 | 4.90 | 5.24 |  | 72.16 | 4.92 | 5.26 |
| **3b** | C_16_H_10_F_3_NOS (321.32) | 59.81 | 3.14 | 4.36 |  | 60.04 | 3.15 | 4.37 |
| **3c** | C_13_H_13_NO_2_S (247.31) | 63.13 | 5.30 | 5.66 |  | 63.38 | 5.32 | 5.68 |
| **3d** | C_13_H_10_F_3_NO_2_S (301.28) | 51.82 | 3.35 | 4.65 |  | 52.02 | 3.36 | 4.67 |
| **3e** | C_11_H_11_NOS (205.28) | 64.36 | 5.40 | 6.82 |  | 64.61 | 5.42 | 6.85 |
| **3f** | C_11_H_8_F_3_NOS (259.25) | 50.96 | 3.11 | 5.40 |  | 51.16 | 3.12 | 5.42 |
| **5a** | C_17_H_13_NO_3_S (311.36) | 65.58 | 4.21 | 4.50 |  | 65.84 | 4.22 | 4.52 |
| **5b** | C_14_H_13_NO_4_S (291.32) | 57.72 | 4.50 | 4.81 |  | 57.95 | 4.52 | 4.83 |
| **5c** | C_17_H_15_NOS (281.37) | 72.57 | 5.37 | 4.98 |  | 72.86 | 5.39 | 4.99 |
| **6a** | C_17_H_13_NO_3_S (311.36) | 65.58 | 4.21 | 4.50 |  | 65.84 | 4.22 | 4.52 |
| **6b** | C_14_H_13_NO_4_S (291.32) | 57.72 | 4.50 | 4.81 |  | 57.95 | 4.52 | 4.83 |
| **6c** | C_17_H_15_NOS (281.37) | 72.57 | 5.37 | 4.98 |  | 72.86 | 5.39 | 4.99 |
| **8** | C_21_H_22_NO_6_PS (447.44) | 56.37 | 4.96 | 3.13 |  | 56.59 | 4.98 | 3.14 |
| **9** | C_21_H_22_NO_6_PS (447.44) | 56.37 | 4.96 | 3.13 |  | 56.59 | 4.98 | 3.14 |
| **10a** | C_24_H_22_NO_5_PS (467.47) | 61.66 | 4.74 | 3.00 |  | 61.90 | 4.76 | 3.01 |
| **10b** | C_19_H_20_NO_5_PS (405.40) | 56.29 | 4.97 | 3.45 |  | 56.51 | 4.99 | 3.46 |
